# Evaluation of Toxicogenomics Approaches for Assessing the Risk of Nongenotoxic Carcinogenicity in Rat Liver

**DOI:** 10.1371/journal.pone.0097678

**Published:** 2014-05-14

**Authors:** Johannes Eichner, Clemens Wrzodek, Michael Römer, Heidrun Ellinger-Ziegelbauer, Andreas Zell

**Affiliations:** 1 Center for Bioinformatics Tuebingen (ZBIT), University of Tuebingen, Tübingen, Germany; 2 Global Early Development, Bayer Pharma AG, Wuppertal, Germany; University of Bonn, Bonn-Aachen International Center for IT, Germany

## Abstract

The current gold-standard method for cancer safety assessment of drugs is a rodent two-year bioassay, which is associated with significant costs and requires testing a high number of animals over lifetime. Due to the absence of a comprehensive set of short-term assays predicting carcinogenicity, new approaches are currently being evaluated. One promising approach is toxicogenomics, which by virtue of genome-wide molecular profiling after compound treatment can lead to an increased mechanistic understanding, and potentially allow for the prediction of a carcinogenic potential via mathematical modeling. The latter typically involves the extraction of informative genes from omics datasets, which can be used to construct generalizable models allowing for the early classification of compounds with unknown carcinogenic potential. Here we formally describe and compare two novel methodologies for the reproducible extraction of characteristic mRNA signatures, which were employed to capture specific gene expression changes observed for nongenotoxic carcinogens. While the first method integrates multiple gene rankings, generated by diverse algorithms applied to data from different subsamplings of the training compounds, the second approach employs a statistical ratio for the identification of informative genes. Both methods were evaluated on a dataset obtained from the toxicogenomics database TG-GATEs to predict the outcome of a two-year bioassay based on profiles from 14-day treatments. Additionally, we applied our methods to datasets from previous studies and showed that the derived prediction models are on average more accurate than those built from the original signatures. The selected genes were mostly related to *p53* signaling and to specific changes in anabolic processes or energy metabolism, which are typically observed in tumor cells. Among the genes most frequently incorporated into prediction models were *Phlda3*, *Cdkn1a*, *Akr7a3*, *Ccng1* and *Abcb4*.

## Introduction

A crucial step in the development of drug candidates is the early exclusion of compounds with carcinogenic potential. The current standard for cancer risk assessment is the two-year rodent bioassay, which involves lifelong treatment of mice and rats at different dose levels. In addition to high costs and sizeable animal use, this method is also known to give false-positive results with respect to human relevance [1]. These may for instance arise from spontaneously formed tumors, from rodent-specific modes of carcinogenicity which do not exist in humans, or from toxic doses causing cell injury or death followed by regenerative cell proliferation [2].

Since the liver is a major target for drug-induced tumor formation, the assessment of cancer risk in this organ is of major importance. A wide variety of mechanisms have been proposed so far for hepatocarcinogenesis. In general, a major distinction is made between genotoxic carcinogens (GC), which induce tumors by direct chemical interaction with DNA, and nongenotoxic carcinogens (NGC), which lead to tumor formation by other mechanisms, such as peroxisome proliferation or secretion of growth-stimulating hormones [3]. For the detection of genotoxicity, a cost-effective short-term test battery, consisting of the Ames test, mouse lymphoma assay, *in vitro* micronucleus or chromosomal aberration test, is routinely performed in the pharmaceutical industry. However, this battery of tests is associated with a high number of false positives [4]. Furthermore, as opposed to GCs, no established short-term toxicity assays exist for the early identification of NGCs. Thus, there is a great demand for the development of reliable prediction methods, and toxicogenomics may be one method which is worth considering in this respect.

Nuwaysir *et al.* first introduced the concept of toxicogenomics and developed a custom cDNA microarray for the extraction of toxicant signatures, i.e., sets of informative genes which are commonly and uniquely differentially expressed upon treatment with toxicants belonging to a certain class [5]. The authors proposed that on the basis of confidently labeled training compounds generalizable models can be constructed that facilitate the classification of unknown test compounds. Furthermore, putative mechanisms of action can be deduced from the observed gene expression patterns.

Various bioinformatics approaches have been developed for the analysis of gene expression profiles induced by treatment with toxic substances. Published approaches for the inference of informative gene signatures include statistical as well as machine learning-based methods. Statistical methods, such as analysis of variance (ANOVA), mixed linear models, principal component analysis (PCA) or Golub’s signal-to-noise ratio (Golub-Ratio) have been successfully adopted for the extraction of genes which are differentially expressed between compound classes [6]–[9]. The so far applied machine learning-based approaches include prediction analysis for microarrays (PAM), Support Vector Machines (SVM), weighted voting, recursive feature elimination (RFE), and other supervised learning algorithms which can be employed for both feature selection and class prediction [6], [9]–[11]. A detailed review of the different approaches used in toxicogenomics studies was recently published by Afshari *et al.* [12]. Notably, in recent years much attention has been drawn towards the microarray-based prediction of nongenotoxic carcinogenesis in rat liver (reviewed by Waters *et al.* [1]).

A large catalogue of transcriptomics datasets is now available in public databases (e.g., Gene Expression Omnibus and ArrayExpress). Furthermore, recently conducted large-scale studies resulted in the assembly of databases specializing in toxicogenomics, such as DrugMatrix (https://ntp.niehs.nih.gov/drugmatrix) and Open TG-GATEs (http://toxico.nibio.go.jp) [13]. Interestingly, regulatory agencies recognize toxicogenomics as a powerful tool to assist in solving toxicologal issues. Both DrugMatrix and TG-GATEs were pointed out as useful resources to gain further knowledge for the drug discovery process [14].

In contrast to universal databases such as Gene Expression Omnibus, these two databases offer organized *in vivo* and *in vitro* datasets from a large set of compounds, which were generated using consistent study designs and standardized experimental protocols to achieve reproducibility and comparability [14]. Both databases comprise time-series measurements of gene expression at multiple dose levels, which were profiled using Affymetrix or Codelink (DrugMatrix only) platforms. For phenotypic anchoring, data from various measurements characterizing the compounds' pharmacology and toxicological effects, including clinical chemistry and histology data, can be downloaded along with the gene expression data.

Here we present two novel approaches for the inference of robust and generalizable gene expression signatures and evaluate their performance with respect to the prediction of a carcinogenic potential, usually assessed in a two-year study, based on public microarray data from various short-term studies. Our first approach employs an ensemble of common feature selection methods for the extraction of informative genes from multiple subsamplings of the training compounds. By considering many variations of the training set and adopting multiple selection algorithms, this approach aims at increasing the generalizability of the inferred signature in the sense that it allows a more reliable identification of NGCs among compounds with unknown carcinogenic potential.

The second approach, which requires less computational power and a smaller amount of training compounds, is based on a moderated signal-to-noise ratio. While traditional approaches select genes differentially expressed between two equally treated classes of samples, our method allows for the extraction of gene expression patterns which are truly specific to the class of primary interest. Thus, we consider our approach better suited for the objective evaluated here, where genes showing a specific response pattern upon NGC treatment and not upon exposure to non-carcinogens shall be identified. On the basis of Golub’s signal-to-noise ratio and the *t*-shrink statistic proposed by Opgen-Rhein and Strimmer, we devised the Specificity Ratio (SR) which detects genes that are specifically deregulated upon treatment with NGCs, while no or only minor expression changes can be observed for NCs [15], [16].

When compared against previously reported signatures on public toxicogenomics data from six previously conducted studies and another dataset obtained from TG-GATEs, we found that on average a higher accuracy could be achieved by classifiers trained on predictive signatures from our Ensemble Feature Selection (EFS) method. We also assessed the classification performance of different ensembles of feature selection methods and investigated the robustness of each individual gene ranking technique in these ensembles.

## Methods

### Ethics Statement

The here analyzed data is publicly available from the TG-GATEs database (http://toxico.nibio.go.jp/english/), which has been established during the Japanese Toxicogenomics Project (TGP).

### Microarray Dataset

The Affymetrix dataset used for classifier training and evaluation was compiled on the basis of the recently published TG-GATEs database [13]. We selected 2 GC, 9 NGC and 11 NC compounds which could be unambiguously assigned to one of these three compound classes. Experimental evidence for class membership was obtained from genotoxicity tests and long-term animal studies (Table 1). The three compounds Methapyrilene hydrochloride (MP), Pirinixic acid (WY) and Monocrotaline (MCT) were considered as undefined, as these showed characteristics of both genotoxic and nongenotoxic compounds.

**Table 1:** Selected compounds from TG-GATEs database.

Class	Compound	CAS Number	Vehicle	Dose [mg/kg/d]	Group ID
**Genotoxic carcinogens**	2-Acetamidofluorene	53-96-3	MC	30	AAF_LD
	N-Nitrosodiethylamine	62-75-9	MC	10	DEN_MD
**Nongenotoxic carcinogens**	Carbon tetrachloride	56-23-5	CO	300	CCL4_HD
	Phenobarbital	50-06-6	MC	100	PB_HD
	Clofibrate	637-07-0	CO	300	CFB_HD
	Hexachlorobenzene	118-74-1	CO	100	HCB_MD
	Phenytoin	57-41-0	MC	600	PHE_HD
	Coumarin	91-64-5	CO	150	CMA_HD
	Ethinyl estradiol	57-63-6	CO	1	EE_LD
	Fenofibrate	49562-28-9	MC	100	FFB_MD
**Non-carcinogens**	Aspirin	50-78-2	MC	450	ASA_HD
	Diclofenac sodium	15307-79-6	MC	10	DFNa_HD
	Ciprofloxacin hydrochloride	93107-08-5	MC	1000	CPX_HD
	Metformin hydrochloride	1115-70-4	MC	1000	MFM_HD
	Nifedipine	21829-25-4	MC	1000	NIF_HD
	Enalapril maleate	76095-16-4	MC	600	ENA_HD
	Mexiletine	5370-01-4	MC	400	MEX_HD
	Triazolam	28911-01-5	MC	1000	TZM_HD
	Meloxicam	71125-38-7	MC	30	MLX_HD
	Lornoxicam	70374-39-9	MC	3	LNX_HD
	Cyclosporine A	59865-13-3	CO	100	CSA_HD
**Undefined compounds**	Methapyrilene hydrochloride	135-23-9	MC	100	MP_HD
	Wy-14643	50892-23-4	CO	10	WY_LD
	Monocrotaline	315-22-0	MC	10	MCT_MD

The table lists the compounds from the TG-GATEs database which were included into our computational analysis of NGC-specific expression profiles. For each compound CAS numbers are provided as a reference. According to the annotation files from TG-GATEs, either corn oil (CO) or methyl cellulose (MC) were used as vehicles for the administration to rats. From the three dose levels available at TG-GATEs, we selected for each compound individually the dose level on the basis of the tumorigenic dose rate 50 (TD_50_) known from published animal studies. The liver samples of 3 Sprague-Dawley rats were taken for one group. The corresponding IDs include the compound short name as well as the selected dose. Low Dose (LD) = 1/8 of LD_50_, Medium Dose (MD) = 1/4 of LD_50_, High Dose (HD) = 1/2 of LD_50_.

For equipotent dose selection, we considered the doses which were tested in long-term animal studies and finally resulted in the development of first hepatocellular carcinomas. In parallel, we investigated the TD_50_ rates, i.e., the compound dose that leads to tumor formation in half of the tested animals, published in the CPDB database (http://potency.berkeley.edu/). Furthermore, we compared histopathology and clinical chemistry data between different compound administrations within the TG-GATEs database. Altogether, from the three dose levels available for each compound in the database, we selected for each compound individually an appropriate dose which corresponds to 5–30-fold of the TD_50_ rate. Since dose range finding studies up to 14 days are routinely performed for drug candidates in the pharmaceutical industry, this time point was of major practical interest.

### Download and Preprocessing of the Microarray Data

The Affymetrix raw data (CEL files) and the corresponding metadata were downloaded from the TG-GATEs FTP site (ftp://ftp.dbcls.jp/archive/open-tggates/). After importing the Affymetrix raw data into the R software environment, the data was normalized by using the robust multi-chip average (RMA) method implemented in the *affy* package. In a subsequent annotation step gene symbols and Entrez Gene IDs were assigned to each Affymetrix probeset, based on the corresponding metadata package *rat2302.db* provided by Bioconductor [17], [18].

### Signature Extraction by using an Ensemble of Feature Selection Techniques

In this study we conceived and implemented an approach for the extraction of predictive and generalizable mRNA signatures for the detection of nongenotoxic hepatocarcinogenesis in rat. For the ranking of candidate signature genes we used weights of Support Vector Machines (SVM) with linear kernels, Prediction Analysis for Microarrays (PAM), Recursive Feature Elimination (RFE) and Golub’s Signal-to-noise Ratio (Golub-Ratio) [15], [19]–[21]. However, in principle, any other algorithm producing a complete ranking of the genes depending on their potential to discriminate NGCs from NCs could have been used.

### Optimization of the Number of Informative Genes

Each method was applied on 25 random subsamplings of the data (*bootstrap* samples), each containing 90% of the training data. The remaining 10% of the training data (*out-of-bag* samples) were used for evaluating the classification performance based on the area under the ROC curve. For this purpose, 11 different signature sizes between 2 and 100 genes were defined. Then ROC scores were estimated based on the predictions of a fast KNN classifier trained on each signature obtained for a certain combination of a method, bootstrap and signature size. By averaging ROC scores across methods, bootstraps and cross-validation folds, a performance could be assigned to each signature size. The optimal number of informative genes was then determined by fitting a spline function which describes the relation between signature size and classification performance and computing its analytical maximum.

### Assessment of the Stability

In order to assess the robustness of the signatures depending on the selection method and the number of signature genes, a stability index proposed by Kuncheva was used [22]. Given two feature subsets *A* and *B* of size *k*, which have *r* features in common and which were selected from a set of *n* features, the Kuncheva index (KI) serves as a measure for the consistency between *A* and *B* and is formally defined as 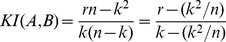 [22].

Obviously, *KI(A,B)* monotonically increases with *r*, the number of features shared between *A* and *B*. The term *k^2^/n* was included to correct for the common selection of features by chance and transforms the KI to a value range between −1 and 1. A value of 0 is expected for independently drawn feature subsets which overlap purely by chance. While negative values of the KI suggest that the selected subsets are more complementary than expected by chance, positive values indicate higher overlaps than expected by chance. The maximum is obtained for two equal subsets and the minimum is reached for two disjoint subsets with *|A| = |B| = n/2.* The KI can also be computed for *m>2* feature subsets by averaging across all pairs of subsets:
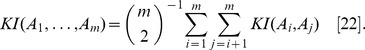



### Merging of the Signatures

In order to obtain a robust consensus signature which is less dependent on the employed selection method and the compounds used for training, the sets of informative genes selected by diverse methods on different bootstraps were merged. For this purpose, the gene ranks obtained from individual bootstraps and methods were summed up. Then the genes were sorted in ascending order based on their rank sums to obtain a consensus ranking. Given the ranking and the previously determined optimal number of informative genes, the final multi-gene signature was generated.

### Specificity Ratio for Signature Extraction

As an alternative to the Ensemble Feature Selection (EFS) approach, which makes extensive use of diverse machine learning algorithms, we conceived a second, purely statistical approach. Our Specificity Ratio (SR) relies on a similar concept as Golub’s ranking method, which selects informative genes, based on high expression differences between classes and small deviations within classes [15]. Golub *et al.* proposed to calculate for each gene 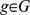 the signal-to-noise ratio 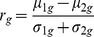, where *µ_1g_* and *µ_2g_* denote the fold-changes of *g* averaged across all samples in class *c_1_* and *c_2_*, respectively. *σ_1g_* and *σ_2g_* denote the corresponding standard deviations [15].

Please note that high positive or negative values of *r_g_* may indicate significant expression differences between the two classes. However, *r_g_* does not ensure that the selected genes have class-specific expression profiles in the sense that high expression changes are only present in the class of primary interest. As we aimed at selecting genes, which are up- or downregulated in the primary class *c_1_* (here: NGC) while being not differentially expressed in the secondary class *c_2_* (here: NC), we replaced 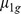 and 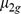 by the average absolute fold-changes 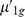 and 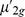 with 
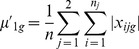
 in the denominator of Golub’s ratio. In this equation 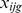 denotes the log_2_(fold-change) of a gene *g* in the *i*-th compound of class *c_j_* which contains *n_j_* compounds. The total number of compounds is 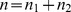.

In the next step, the gene-specific standard deviations *σ_g_* were shrunken towards the median variance *σ_0_* averaged across all genes, in order to avoid the overvaluation of genes with marginal differential expression, due to an underestimation of the sample variance. For this purpose, we replaced the gene-specific standard deviations *σ_g_* by the moderated standard deviations 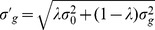 used by Opgen-Rhein and Strimmer in their *shrink-t* statistic [16]. The authors proposed to adjust the variances by using a weighed sum of the gene-specific variances 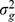 and the overall variances 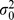, where the weight of 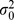 is given by a pooling parameter *λ* with 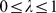.

The optimal value for *λ* is determined based on the variances 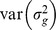 of the gene-specific variances 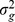 as defined in the following equations:
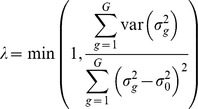





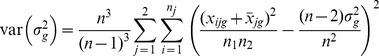



Intuitively, the main idea is that if the variance 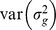 of the gene-specific variances 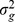 is high, these values could not be reliably obtained from the data and thus, should be adjusted by shrinkage towards the target 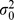 [16]. Otherwise, if all 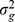 could be reliably estimated with small variance 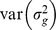, the adjustment should be smaller. The complete mathematical derivation of the formulas used for the estimation of the moderated standard deviations 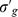 was provided earlier by Opgen-Rhein and Strimmer [16]. Finally, on the basis of Golub’s ratio 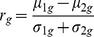, we defined the *specificity ratio* 
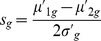
.

### Post-Filtering of Candidate Signature Genes

Since our ratio *s_g_* is based on the absolute values of the fold-changes, the direction of a gene’s deregulation may vary across the compounds in the primary class *c_1_*. In order to filter genes which are either consistently up- or downregulated, we applied a ROC-based filter to the obtained gene lists. This filter computes a ROC score for each gene by comparing the fold-changes observed for the different compounds to their corresponding compound class. The ROC score reaches its maximum (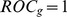) for a particular gene *g*, if the fold-changes measured for the compounds of the primary class (here: NGC) are all greater than the fold-changes observed for the secondary class (here: NC). For an equally informative gene which is consistently more strongly downregulated upon treatment with primary class compounds, the ROC score would be 0. If there is no correlation between gene expression and the compound class, we would expect a ROC score of 0.5. As we aimed at filtering both consistently up- and downregulated genes, we computed for each gene 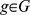 the maximum ROC score 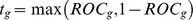 obtained for regular and inverted class labels, respectively. Then we used a cutoff of 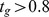 to filter relevant signature genes.

### Assessment of the Classification Performance

Ultimately, the selected informative genes were incorporated into different prediction models which were evaluated on independent sets of test compounds. To this end, we trained 6 different classifiers based on the learning algorithms SVM, KNN, PAM, Random Forest, Weighted Voting and Naive Bayes and evaluated the performance of these classifiers based on a nested 3×3-fold cross-validation [15], [19], [21], [23]. Within each fold of the outer cross-validation, an inner 3-fold cross-validation was performed on the corresponding training data to determine the optimal model parameters.

### Scaling of Classifier Inputs and Outputs

The standardized fold-changes (i.e., *z*-scores) of the informative genes were used as features for model construction. Specifically, for each gene the mean *μ* and the standard deviation *σ* of the fold-changes were calculated and then each fold-change was transformed into a *z*-score 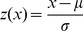. For the sake of simplicity and better comparability, the prediction scores returned by the classifiers were mapped to a value range between 0 and 1. As SVM outputs were contained in the interval 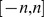, where *n* varies between models depending on the used training data and hyperparameter *C*, the sigmoidal function 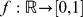 with 
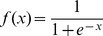
 was used for scaling. The confidences obtained from Weighted Voting classifiers, which originally ranged between −1 and 1, were transformed using the linear function 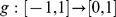 with 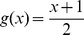. For KNN we computed continuous outputs, based on the function 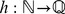 with 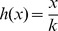 where *k* denotes the number of nearest neighbors and *x ≤ k* is the number of positively labeled ones among these.

### Pathway Analysis

Enrichment analysis against KEGG pathways was performed to investigate putative mechanisms of action [24]. Overrepresentation of genes from specific pathways among the signature genes was determined by a hypergeometric test. Given a set of *n* signature genes of which *m* are contained in a certain pathway and provided that the union of all pathways contains *N* genes of which *M* are in the considered pathway, the p-values were computed according to the following formula:
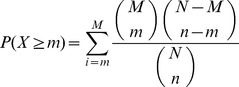



The resulting p-values were corrected for testing multiple pathways using the FDR method by Benjamini and Hochberg [25]. As criterion for significant enrichment we used a p-value cutoff of 0.05.

### Implementation

All plots shown in the results section were generated using the R programming language for statistical computing and packages from the Bioconductor library [17]. For SVM training and classification the *libsvm* implementation from the SHOGUN toolbox was used [19]. RFE was implemented in R on top of the linear kernel SVM from SHOGUN [19]. Golub-Ratio and Weighted Voting were implemented from scratch based on the description of the algorithm from Golub *et al.* [15]. For KNN, PAM, Naive Bayes and Random Forest the R packages *knnflex*, *pamr*, *klaR* and *randomForest* were used [21], [23]. In order to assess the prediction accuracy of the classifiers ROC curves were generated using the *ROCR* package [26].

## Results

**Figure 1:**
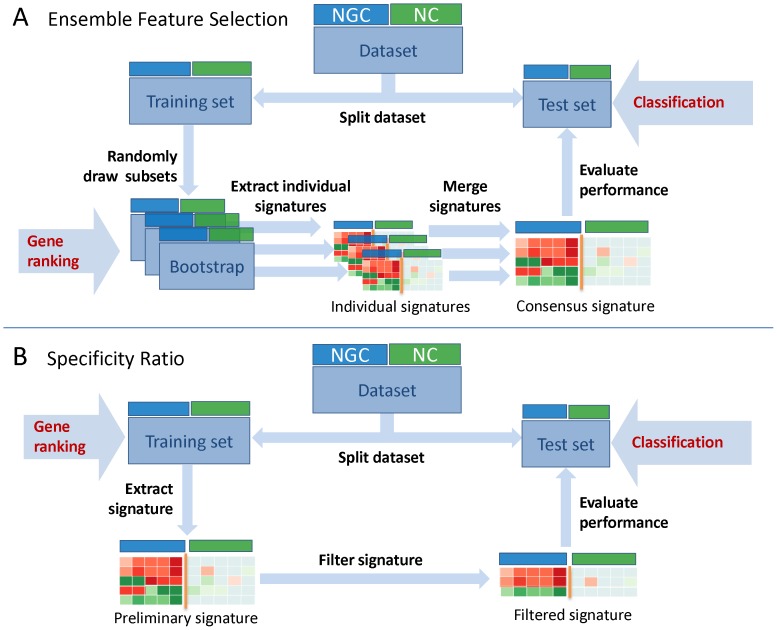
Methodologies used for signature extraction and compound classification. (**A**) Ensemble Feature Selection method: First, the compounds are subdivided into a training set and a test set. Then *n* different subsamplings (bootstraps) each containing 90% of the training compounds are randomly drawn. Gene rankings are generated on each bootstrap by *m* different algorithms and *n×m* signatures are inferred. The individual signatures are subsequently merged and incorporated into diverse classifiers which are applied to the test compounds in order to assess the performance. (**B**) Specificity Ratio method: After splitting the dataset, the gene ranking is directly performed on the training compounds. A preliminary signature is generated from a gene ranking according to the specificity ratio. Then genes exhibiting inconsistent expression profiles across the compounds of the primary class are removed using a ROC-based filter. Finally, the performance of the filtered signature is evaluated on the test set.

In this study we introduce and compare two novel feature selection methods which were specifically designed for the problem of extracting informative genes for NGC/NC classification from gene expression data (Figure 1). First, the performance of both the machine learning-based EFS and the statistical SR method was assessed on a microarray dataset obtained from the Japanese toxicogenomics database TG-GATEs. For the EFS method, we also evaluated the performance achieved with different ensembles of feature selection techniques and assessed the stability of the selected informative genes for each gene selection method individually. The two proposed methodologies were then compared against seven signatures reported in previous studies. Since except for one study the data has been deposited in the public domain, we also evaluated our methods against six of the previously reported signatures on the original datasets. Finally, we applied the methods to predict the carcinogenic class of the compounds MCT, MP and WY which showed characteristics of both genotoxic and nongenotoxic compounds in published carcinogenicity studies.

### Classification Performance Achieved with Novel EFS and SR Method

The standard gene selection methods Golub-Ratio, SVM, PAM and RFE as well as the statistical inference methods t-test, Wilcoxon rank-sum test, and permutation test were employed to infer predictive signatures for the reliable discrimination of NGCs from NCs, based on Affymetrix data from Sprague-Dawley rats treated for 14 days in a repeated dosing study. Next, a consensus signature was compiled by integrating the individual gene rankings obtained from diverse ensembles of gene selection methods across varying subsamplings of the training data (bootstraps). The idea of this approach is firstly, that the integrated signature contains only genes which were deemed informative by multiple independent selection methods. Secondly, it is characterized by increased robustness against variations in the set of training compounds, as the respective genes were highly ranked for multiple random subsamplings of the data. We inferred predictive signatures based on three different variants of our EFS methodology, which involve the use of standard gene selection methods, the statistical inference methods t-test, Wilcoxon rank-sum test, and permutation test, or both types of ranking algorithms. Depending on the employed ensemble of methods the resulting signature size ranges between 36–54 probesets referring to 24–45 annotated genes (Table S1).

A second signature was compiled by selecting the 100 genes with the strongest NGC-specific expression pattern according to our Specificity Ratio. As described in more detail in the methods section we refined the signature by using a ROC-based filter, which excludes genes showing inconsistent regulation states upon NGC treatment. The final SR signature contains 29 probesets which are attributed to 24 genes (Table S1).

**Figure 2:**
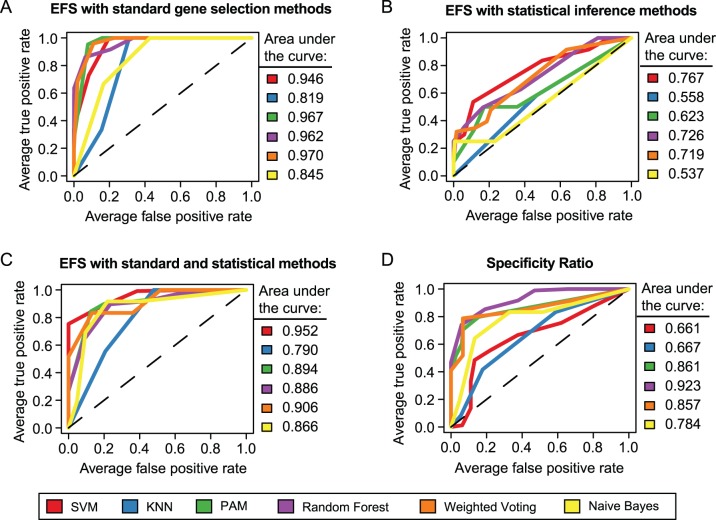
Evaluation of EFS-based and SR-based signatures on TG-GATEs data. The ROC curves obtained from different cross-validation folds were averaged based on the thresholds for class discrimination and drawn separately for each of the six classification methods. The classifiers evaluated here were trained on features selected using our (**A–C**) EFS methodology in conjunction with (**A**) the standard gene selection methods Golub-Ratio, PAM, SVM and RFE, (**B**) the statistical inference methods t-test, Wilcoxon rank-sum test and permutation test or (**C**) all previously stated methods. (**D**) The prediction accuracy was also determined for the SR signature-based models and the corresponding ROC curves were generated as described previously.

We evaluated the classification performance by incorporating each of our signatures into prediction models, which were constructed using established supervised learning algorithms. For each classifier an averaged ROC curve was generated based on the results from a 3×3 nested cross-validation (Figure 2). Apparently, the performance of the EFS method heavily depends on the employed ensemble of feature selection techniques. While depending on the adopted classifier good or excellent classification accuracy was found for standard gene selection methods, the performance achieved with t-test, Wilcoxon rank-sum test, and permutation test was considerably weaker (Figure 2A, B). By combining both standard methods for gene ranking and the three evaluated statistical methods it was also possible to construct fairly well generalizable prediction models (Figure 2C). However, despite the increased complexity, the models did not improve in terms of prediction accuracy. Therefore, we did not pursue this approach any further. The steep initial slope of the ROC curves shown in Figure 2D indicates that the SR method also permits the specific detection of NGCs at a low false positive rate. However, when compared to the EFS signature, the SR signature is clearly limited in terms of sensitivity, which may in part be owed to the stringent selection of genes with NGC-specific expression patterns. PAM, Weighted Voting and Random Forest were found to be the most reliable classification methods for both the EFS and the SR signature. When compared to these classifiers, KNN and Naive Bayes tend to result in worse prediction accuracies. The SVM classifier performs well in conjunction with the EFS signature, but is not recommended for use with the SR signature.

### Comparison of Feature Selection Methods in Terms of Accuracy and Robustness

Besides assessing the predictive power of EFS-based signatures depending on the applied ensemble of methods, we also evaluated the robustness of each individual method against variations of the training data. For this purpose, we used the stability index KI proposed by Kuncheva (see methods section) [22]. Concurrently, the average performance of the individual signatures inferred from varying subsets of the training data was measured in terms of the ROC scores on the 10% out-of-bag samples, which were excluded from each bootstrap. For both scores the optimum is 1, but the values assigned to the worst cases are different. While a ROC score of 0.5 corresponds to the performance achieved by a random guesser, a KI of 0 would be expected for randomly drawn feature subsets.

**Figure 3:**
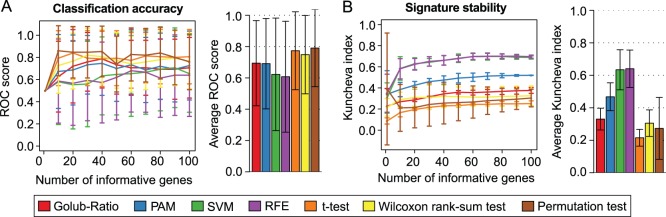
Accuracy and stability of signatures depending on gene selection method and signature size. (**A**) The line plots illustrates the mean classification performance achieved by a KNN classifier based on diverse signatures selected by the methods Golub-Ratio, SVM, PAM, and RFE. The performance was measured in terms of area under the ROC curve for 11 linearly spaced numbers of informative genes ranging from 2 to 100. Points and whiskers correspond to the means and the standard deviations of the ROC scores assessed on the *out-of-bag* samples of 25 bootstraps during 3-fold cross-validation. The adjacent bar plots depict the corresponding ROC scores averaged across signature sizes and cross-validation folds. (**B**) The Kuncheva index (KI) was used to score the correspondence of signatures selected on different bootstraps depending on the selection method and the number of selected genes. The average KI obtained for each method is shown in the bar plot on the right.

The accuracy and stability of the signatures selected by different methods is depicted depending on the number of informative genes in Figure 3. According to the curves in Figure 3A the highest average classification accuracy could be achieved with the methods t-test, Wilcoxon rank-sum test and permutation test, which select informative genes based on statistical inference. For these statistical tests the maximum accuracy was attained with at most 30 informative genes. However, as illustrated in Figure 3B a relatively low consistency was observed between the gene sets selected on different bootstraps. Similarly, a relatively low signature stability was found for Golub’s ranking method, which also relies on a statistical comparison of the classes. Golub’s method reached its maximum accuracy in conjunction with a signature size of 40 (average ROC score: 0.79). The decrease of performance which is observable for increased signature sizes (>40 genes) may be partly due to overfitting, caused by the selection of genes with minor relevance for the problem of distinguishing NGCs from NCs. A marginally weaker performance than for the Golub-Ratio was found for the PAM method, which also reaches its maximum accuracy when only the 40 top ranked genes are used for model construction. However, the informative genes selected by PAM are clearly less dependent on the subsampled part of the training data, which becomes apparent from Figure 3B. Strikingly, a considerably higher robustness against sampling variation was found for the two SVM-based methods. However, the classification accuracy achieved with signatures from SVM and RFE was significantly lower when compared to Golub-Ratio and PAM, especially for smaller signature sizes. A comparable, moderate performance, but significantly increased robustness can only be observed for larger signature sizes, for instance, when 70 informative genes are selected.

### Comparison Against Existing Signatures for NGC Prediction

We then compared our novel signatures to several already available ones which had been derived using widely used supervised classification methods [6], [10], [11], [27]–[30]. Furthermore, we determined which genes have been selected most frequently. Besides emphasizing the most relevant informative genes, we also point out shared mechanistic characteristics, which were identified based on pathway enrichment analysis.

**Figure 4:**
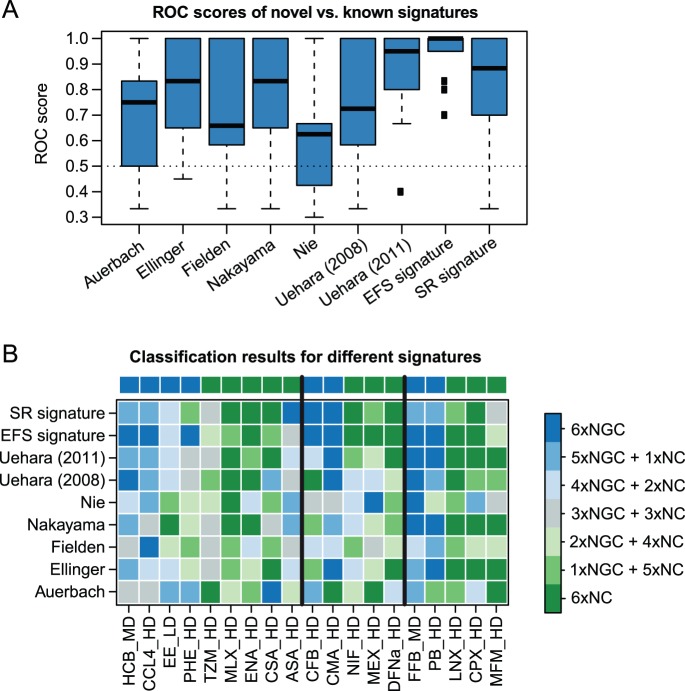
Performance comparison against signatures known from the literature. (**A**) Box plots show the distribution of ROC scores resulting from 3-fold cross-validation of 6 different classifiers incorporating either one of 7 published signatures or one of our 2 novel signatures. Each box corresponds to a certain multi-gene signature. The lower, center, and upper line of each box indicates the lower quartile, median, and upper quartile of the ROC scores obtained for a certain signature. The whiskers extend to the most extreme ROC score which is within 1.5 times the interquartile range from the box. Outliers are depicted as individual points. (**B**) The heatmap shows the binary classification outcomes of 6 predictors used for NGC vs. NC discrimination, depending on the incorporated signature. The colors indicate the number of methods which classified a certain compound as NGC and NC, respectively. Cells are drawn in blue if a compound was classified as NGC and in green if it was classified as NC by the majority of predictors. Grey color indicates a tie between the two classes. In order to discretize the continuous prediction scores, the class discrimination cutoff was chosen individually for each predictor as an optimal tradeoff between sensitivity and specificity.

The classification performance, that has been achieved by 6 representative machine learning methods, was assessed for all signatures using cross-validation and measured based on the area under the ROC curve. The distributions of the resulting ROC scores are illustrated as box plots in Figure 4A. Another illustration, which depicts the predictions made for the individual compounds in more detail, is shown in Figure 4B.

The boxes shown in Figure 4A clearly indicate that the highest average classification performance is achieved by classifiers incorporating our novel 54-probeset EFS signature (mean ROC score: 0.95). In this performance comparison the second best signature was the one proposed by Uehara in 2011 followed by our SR signature (Table 2). The signatures by Ellinger-Ziegelbauer and Nakayama also show a trend towards higher accuracies when compared to the rest. Consistent with our expectations the Uehara (2008) signature was outperformed by the more recent Uehara (2011) signature, as it primarily captures changes in gene expression present after 24 hours upon one-time NGC exposure, which are of minor relevance for the 14-day administration setting evaluated here. Similarly, the low performance found for the Auerbach signature, which was designed for longer treatment durations up to 90 days, must be seen in the context of its limited applicability to the 14-day setting.

**Table 2:** Published and novel signatures for NGC prediction in rat liver.

Signature	Number of probesets	Number of genes	Relevant time point	Microarray platform	Mean ROC score
Auerbach	11	10	90 days	Agilent Whole Rat Genome Microarray	0.69±0.20
Ellinger	101	76	14 days	Affymetrix RAE 230A	0.80±0.18
Fielden	35	27	5 days	Amersham Codelink	0.70±0.22
Nakayama	56	44	28 days	Custom oligonucleotide microarray	0.78±0.23
Nie	7	6	24 hours	Custom cDNA microarray	0.59±0.19
Uehara (2008)	112	101	24 hours	Affymetrix RAE 230 2.0	0.74±0.22
Uehara (2011)	82	68	28 days	Affymetrix RAE 230 2.0	0.87±0.17
EFS signature	54	45	14 days	Affymetrix RAE 230 2.0	0.95±0.09
SR signature	29	24	14 days	Affymetrix RAE 230 2.0	0.83±0.20

The table lists all signatures compared in this study and indicates the respective number of probesets which originate from or were mapped to the Affymetrix Rat Genome 230 2.0 Array. Along with the number of probesets the number of corresponding genes is shown. For each signature the treatment duration and microarray platform used in the original study is specified. The rightmost column contains the mean ROC scores and standard deviations which resulted from evaluation on the TG-GATEs dataset.

**Figure 5:**
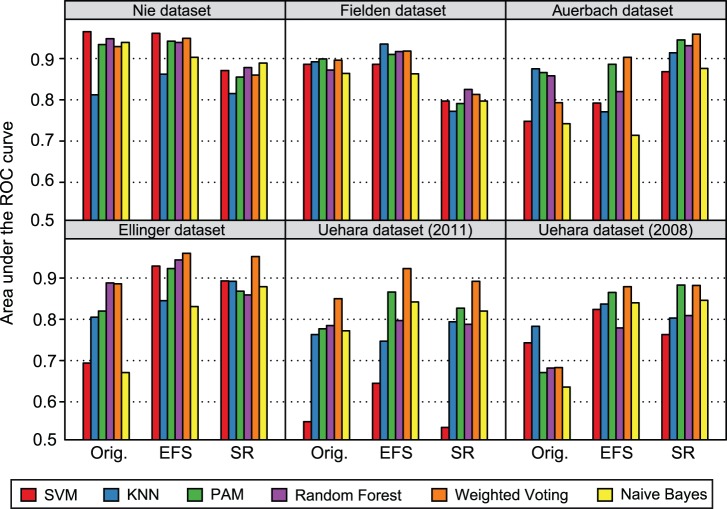
Evaluation of EFS-based and SR-based signatures on datasets from previous studies. The bar plots depict the area under the ROC curve achieved by specific prediction models built and evaluated on datasets which have been used in related toxicogenomics studies. The prediction models differ in the adopted classifiers and in the incorporated signatures. On each dataset, two signatures were extracted using the EFS and SR method, respectively, and compared to the signature from the original study in terms of classification performance. Each dataset corresponds to a certain panel (see panel headers), each signature is represented by a group of bars, and the classifiers are indicated by different colors (see legend).

In order to enable a conclusive and fair comparison to each of the existing signatures, we also applied our approaches to the original datasets from which these signatures were identified. Consistent with our evaluation on the TG-GATEs data, the models constructed from the EFS-based signatures achieved the highest prediction accuracy on 5 of 6 published toxicogenomics datasets, when compared to classifiers trained on the original and SR-based signatures, respectively (Figure 5). The corresponding ROC curves can be found in Figure S1-S6. Only on the dataset compiled by Auerbach *et al.*, the SR-based signature performed better. Considering the average performance across all datasets and signatures, the best classifier was Weighted Voting (mean ROC score: 0.88), followed by PAM and Random Forest.

**Figure 6:**
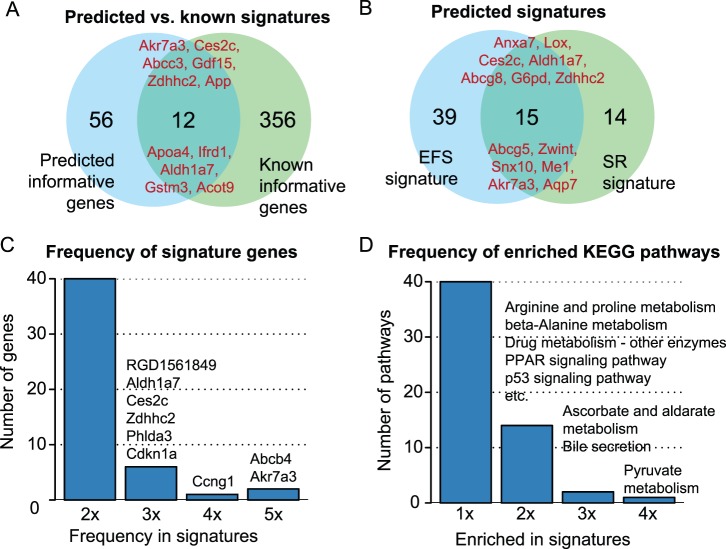
Comparison of predicted and known signature genes. (**A**) The Venn diagram depicts the overlap between the informative genes from the EFS and SR signature and the genes contained in 7 previously reported signatures. The symbols of known marker genes independently confirmed by our approach are listed in the intersection of the two sets. (**B**) This diagram illustrates the overlaps between the two novel signatures predicted using the Ensemble Feature Selection (EFS) method and the Specificity Ratio (SR), respectively. (**C**) The histogram shows the absolute selection frequencies of informative genes which are part of the 2 novel signatures and the 7 previously published ones. The genes which are included in 2 or more signatures are named on top of the corresponding bars. (**D**) For each signature a pathway enrichment analysis against KEGG was performed and then a histogram was generated that shows which pathways were most frequently detected as enriched among the informative genes. Some of the KEGG pathways which were detected to be significantly enriched in two or more signatures are listed on top of the respective bars.

Besides comparing our novel signatures to published ones in terms of classification accuracy, we determined which informative genes were commonly selected (Figure 6A–C). Among these genes is, for instance, *App*, which is a key protein in the pathomechanism of Alzheimer’s disease, but was also reported to be upregulated in different cancer types, and shown to play a crucial role in the growth control of pancreatic and colon cancer [31]. Another highly informative gene is *Cdkn1a*, which is a *p53*-dependent key regulator of cell fate, as it triggers cell cycle arrest in the G1 phase under various stress conditions, such as DNA damage [32]. Mechanistically similar growth-inhibitory effects were reported for *Ccng1*, which is also known to be transcriptionally regulated by *p53*, and which was shown to induce *pRb*-dependent G1 phase arrest when being highly expressed [33]. *Phlda3,* which was proposed as a potential biomarker in diverse toxicogeniomics studies [6], [10], [29], is a known target gene of *p53*, too. *Phlda3* may act as a tumor suppressor by inhibiting the translocation and activation of *Akt,* thereby preventing negative regulation of *p53*-dependent apoptosis via *Akt* [34]. The *Akr7a3* transcript, which is part of our proposed signature and which was mostly found upregulated in response to carcinogen treatment, was previously considered as informative by Ellinger *et al.*, Nakayama *et al.* and Uehara *et al.* [6], [10], [29]. Consistent with these results, in a recent explorative study aiming at the identification of novel candidate biomarkers for liver cancer, an upregulation of *Akr7a3* on the protein level was observed in rat hepatomas [35]. Another frequently used informative gene is *Abcb4* alias *Mdr3*. This multidrug-resistance gene is typically expressed in diverse tumors, rendering them less sensitive to treatment with anti-cancer drugs (Figure 6C) [36].

In addition to determining the most commonly selected informative genes, we also compared the signatures with respect to their associated pathways (Table S2). It can be concluded from Figure 6D that signatures for early NGC detection frequently include genes involved in energy metabolism, anabolic metabolism, drug metabolism (e.g., by CYP enzymes), and DNA-damage response (e.g., via p53 pathway). These changes clearly reflect a response to carcinogens including starting cellular hyperplasia upon treatment. However, it should be noted that the early molecular events captured by the proposed signature genes are not specifically related to nongenotoxic mechanisms, of which a wide variety have been proposed for rat liver [3]. As representing the complete spectrum of NGC mechanisms in one gene list is hardly feasible, most of the current signatures for NGC detection are also sensitive to genotoxic carcinogens (Figure S7).

### Expression Profile Analysis of Undefined Compounds

For three of the analyzed compounds, namely MP, WY and MCT a definite assignment to either GCs or NGCs was not possible based on published animal studies and genotoxicity assays [37]–[39]. As only two unambiguously classified GCs (AAF and DEN) are available from TG-GATEs, we take the view that it is not feasible to construct generalizable toxicogenomics models for reassigning the undefined compounds to either GCs or NGCs. Nevertheless, we compared the expression profiles of these undefined substances to the ones observed for unambiguously classified compounds, and evaluated whether a detection of their carcinogenic potential was possible based on our multi-gene signatures.

**Figure 7:**
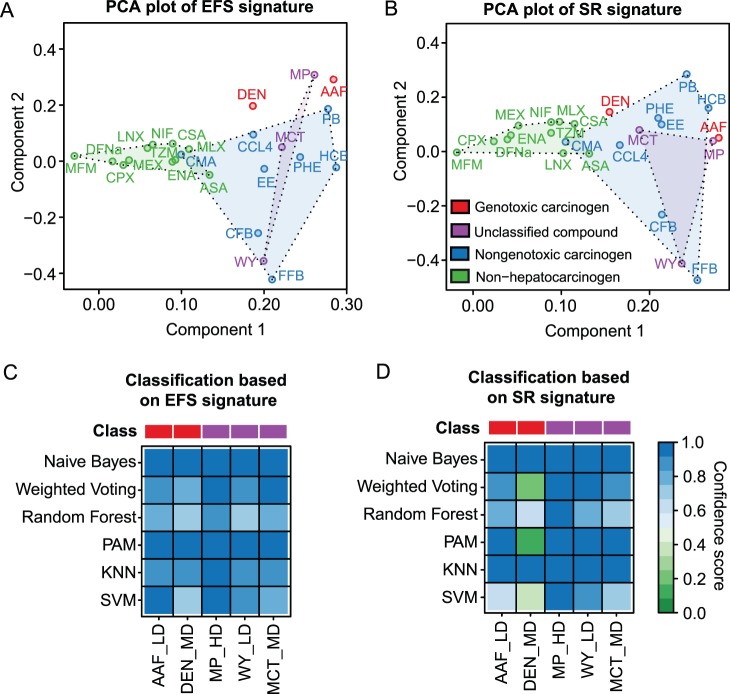
Separation and classification of compounds based on EFS and SR signature. (**A**) The dots correspond to different treatment groups and are colored according to the classes of the compounds used for treatment. Each treatment group was originally represented by a vector composed of the fold-changes of the 54 signature genes measured after 14 days of repeated dosing. In order to inspect the compound-specific expression profiles in a lower-dimensional space, these vectors were transformed to the first and second principal component resulting from PCA. In order to highlight clusters of NGCs and NCs, convex hulls were drawn around the respective compounds. The compounds WY, MP and MCT were considered as undefined, due to ambiguous outcomes of published studies. (**B**) PCA plot similar to (A), but generated on the basis of the SR signature. (**C**) The heatmaps depict the confidence of the predictions made by diverse classifiers for assessing the carcinogenic potential of GCs (AAF, DEN) and undefined compounds (MP, WY, MCT). Columns represent compounds and rows correspond to classifiers. The compound classes are indicated by the colorbar on top. The discrimination between carcinogens (blue) and non-carcinogens (green) was done based on the EFS signature. (**D**) Toxicogenomics-based assessment of the carcinogenic potential of GCs and undefined compounds using diverse classifiers which incorporate the SR signature genes as predictive features.

**Figure 8:**
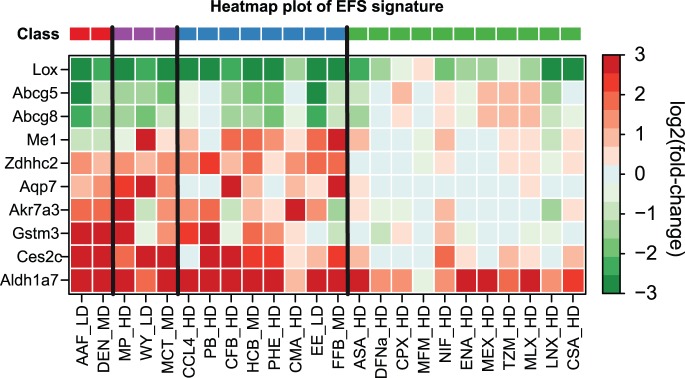
Expression profiles of EFS signature genes. Shown is a heatmap depicting the fold-changes of the top 10 informative genes from the predicted EFS signature. Rows represent genes and columns represent treatment groups. Cell colors indicate the strength and direction of differential expression relative to the corresponding control groups (red: upregulation, green: downregulation). Treatment groups which belong to different compound classes are separated by solid vertical lines. The respective classes are indicated by the color bar on top of the heatmap.

In order to generate a graphical representation of the compounds’ expression profiles, the dimensionality of our predicted signatures was reduced to a two-dimensional space using PCA (Figure 7A–B). Obviously, a clear separation between NGC- and NC-treated sample groups is possible, based on both the EFS and the SR signature after 14 days of repeated dosing. While for most published signatures considerable overlaps could be observed between the NGC and the NC clusters (Figure S8), only a small overlap, caused by the compound CMA, was found for the EFS and SR signature, respectively. Consistent with this observation, the expression changes in the top 10 genes of our EFS signature (Figure 8) and SR signature (Figure S9) are less pronounced for CMA when compared to the other NGCs. It also becomes apparent from Figure 8 that the expression differences between NGCs and NCs are much more striking than the differences between NGCs and GCs. This finding is consistent with the fact, that the EFS signature is also sensitive to GCs. In general, it can be concluded that owing to the circumstance that the GC class was not considered during informative gene selection, the inferred signatures are not specifically related to nongenotoxic mechanisms, but rather to hepatocarcinogenesis in general. Nevertheless, individual informative genes (e.g., *Me1*) exist, that are clearly differentially expressed between GC and NGC (Figure 8), which may in part explain the isolated positions of the genotoxic compounds DEN and AAF in PCA space (Figure 7A).

In the PCA plots the three undefined compounds MP, WY and MCT were found to be very distinctly positioned (Figure 7A–B), which indicates compound-specific differences in the expression profiles of the selected informative genes. While the profile of MP resembles the one of the genotoxic compound AAF, WY is located near the nongenotoxic substances CFB and FFB (Figure 7A–B). MCT also tends to show a rather NGC-like profile similar to the ones observed for CCL4, PHE, and EE. Whereas a certain similarity between the profile of WY and those of other NGCs was also apparent from PCA representations obtained from most published signatures (Figure S8), this was not the case for MCT which mostly clustered in the vicinity of the genotoxic compound DEN.

## Discussion

In this study, we evaluated two alternative methodologies for the extraction of predictive molecular signatures from toxicogenomics datasets to allow classification of compounds with respect to their chronic effects based on short-term expression profiles. In contrast to most previous approaches, which employed individual selection algorithms on a fixed set of compounds, our EFS method integrates the results from an ensemble of feature selection techniques applied to multiple randomly drawn subsets of the training compounds. We designed this methodology under the assumption that genes which are truly relevant for compound classification will have a higher chance to be repeatedly selected by independent methods. Furthermore, bootstrapping was used to increase the robustness of the signature in the sense that it is insensitive to small variations of the training data. Our EFS method builds on concepts which were theoretically described by He *et al.* and practically applied by Abeel *et al.*, who implemented an SVM-based method for the discrimination of diverse cancer types [40], [41]. In contrast to the method proposed by Abeel *et al.*, our signature extraction method is not intrinsically multivariate, as it does not score the joint predictivity of a set of genes, but selects genes based on a high average rank assigned by independent methods. Furthermore, our EFS method is capable of optimizing the signature size by fitting a non-linear model which describes the classification performance depending on the number of informative genes.

The SR method was derived from a gene ranking method proposed by Golub *et al.* [15]. The main idea of Golub’s signal-to-noise ratio is to preferentially select genes which show high expression differences between classes, while at the same time their variation within classes is small. Occasionally, this method tends to select inappropriate genes, due to an underestimation of the variances observed within classes. In order to overcome this problem, we used moderated standard deviations as proposed by Opgen-Rhein and Strimmer in their shrink-t statistic [16]. Another problem which was inadequately considered by most previous approaches is the fact that ordinary feature selection algorithms treat both classes equally. In the here evaluated toxicogenomics setting, this may result in the counterproductive selection of genes which are deregulated in NCs while remaining unchanged in NGCs. We avoid this problem by using the difference of the mean absolute fold-changes between a primary and secondary class in the numerator of Golub’s ratio. However, as this modification may cause the selection of informative genes which are not consistently up- or downregulated, an additional post-filtering step is required. In contrast to the SR method the performance of most machine learning-based methods depends on the proper selection of specific parameters, which require optimization on an additional validation set or by nested cross-validation. As the SR method does not require an additional set of compounds for model selection, it is particularly suited for toxicogenomics applications, where typically only few compounds are available for training.

In a performance comparison against 7 previously reported informative gene sets for NGC detection, we demonstrated that the signatures inferred by our EFS and SR method enable 6 state-of-the-art classifiers to achieve comparably high prediction accuracy. Especially the EFS signature stands out as the gene set for which by far the highest classification performance was observed. This finding was also substantiated by the fact that EFS-based signatures permitted the construction of the most accurate prediction models on the majority of datasets used in previous studies.

When comparing different variants of the EFS method, the best performance was found for an ensemble with the traditional gene selection methods Golub-Ratio, PAM, SVM and RFE. These feature selection techniques were also shown to produce more consistent signatures than the statistical inference methods t-test, Wilcoxon rank-sum test, and permutation test, when applied to varying subsets of the training data. Since the inference of a consensus signature is more straightforward given a homogeneous set of signatures, this favorable characteristic may partly explain the suitability of these methods for an EFS approach.

Furthermore, we would like to point out that the viability of the EFS and other omics-based approaches for the prediction of chronic toxicity also depends on the effective treatment of experimental artifacts, such as batch effects. In this study, the application of the RMA algorithm was sufficient to correct for experimental variation. However, if systematic non-biological effects persist despite application of appropriate normalization methods, we recommend the use of specifically designed software for the removal of batch effects (e.g., SVA package for R/Bioconductor) [42], [43].

As fairly reliable short-term tests exist for the prediction of GCs, we focused on NGC detection in this study. To this end, we carefully selected suitable training compounds from the TG-GATEs database, which can be unambiguously classified as NGCs based on experimental evidence from published studies. Despite of the fact that exclusively NGCs were used for training, our proposed EFS signature and to a lesser degree also the SR signature were demonstrated to be also suited for the detection of GCs. Additionally, the sensitive detection of the undefined compounds MP, MCT and WY, which show characteristics of both genotoxic and nongenotoxic mechanisms, renders proof of the good generalizability of the two signatures.

WY was originally classified as an NGC which can be further characterized as a peroxisome proliferator [44], [45]. Consistent with this view, experimental evidence for WY acting as an NGC was obtained from a negative Ames test [46]. However, another *in vitro* test, which is based on single cell gel electrophoresis, revealed that WY also induced DNA strand breaks in this setting, as typically observed for GCs [37]. Despite a negative Ames test result for MP, a potential mutagenic action of this compound was observed in the mouse lymphoma assay [38]. On the other hand, a genotoxic mRNA signature induced by NGCs in liver may also arise from secondary DNA damage which has been suggested to be caused by an imbalance between cell growth and cell death [47], or which may result from oxidative damage [48]. Similar results were observed for MCT, which was characterized as a nongenotoxic substance, although genotoxicity and morphological changes were detected in a human brain tumor cell line [39].

As cell growth and proliferation in tumors requires increased uptake of nutrients (e.g., glucose), which are required for production of high metabolites and reducing (e.g., ATP, NADH) or macromolecules (e.g., proteins, nucleic acids), drastic changes in the intermediary metabolism can be observed in tumor cells [49], [50]. Since this metabolic reprogramming is mediated via transcriptional regulation these changes also become apparent when monitoring global gene expression. Accordingly, we could find pathways related to energy metabolism and anabolic processes overrepresented among different informative gene sets used for microarray-based identification of NGCs. Another typical response which is captured by transcriptional signatures is the response to DNA damage via the *p53* signaling pathway [51], [52]. While this response is characteristic for genotoxic compounds, which by definition directly interact with DNA, abnormal DNA integrity can also be caused by secondary mechanisms (e.g., oxidative damage via peroxisome proliferation) triggered by NGCs [3], [53], as alluded to above.

This finding may also in part explain the fact that many reported signatures do not exclusively comprise genes which are specifically deregulated upon NGC treatment, and thus require additional short-term toxicity assays (e.g., Ames test) for proper distinction from GCs. In principle, the toxicogenomics-based discrimination of NGCs from GCs could be pursued as a complementary approach. However, due to the lack of reasonable numbers of unambiguously classified GCs in TG-GATEs, which are needed for model construction and evaluation, such an analysis could not be performed here. Concerning this GC-NGC discrimination resources available from other public toxicogenomics databases, such as DrugMatrix could be exploited in future studies. Furthermore, in addition to the commonly performed genome-wide analysis of mRNA expression, other omics datasets, capturing genomic and epigenomic features (e.g., miRNA expression and DNA methylation) on a global level, should be collected, since they can be expected to deliver additional biomarker signatures in association with further insight into responses to carcinogens at the molecular level.

In conclusion, this work adds to the current repertoire of toxicogenomics methodologies for the extraction of predictive signatures from omics datasets, which delivers additional mechanistic insight and can be used, for instance, to prioritize compounds for long term carcinogenicity assays.

## Supporting Information

Figure S1 **Evaluation of EFS-based and SR-based signatures on dataset from Ellinger** ***et al.*** The ROC curves obtained from different cross-validation folds were averaged based on the thresholds for class discrimination and drawn separately for each of the six classification methods (SVM, KNN, PAM, Random Forest, Weighted Voting and Naive Bayes). These classifiers were trained on **(A)** the original signature reported by the authors, **(B)** the signature inferred using our EFS method or **(C)** the signature obtained from our SR method.
(PDF)

Figure S2 **Evaluation of EFS-based and SR-based signatures on data from Uehara** ***et al.*** **(2011).** The ROC curves obtained from different cross-validation folds were averaged based on the thresholds for class discrimination and drawn separately for each of the six classification methods (SVM, KNN, PAM, Random Forest, Weighted Voting and Naive Bayes). These classifiers were trained on **(A)** the original signature reported by the authors, **(B)** the signature inferred using our EFS method or **(C)** the signature obtained from our SR method.
(PDF)

Figure S3 **Evaluation of EFS-based and SR-based signatures on data from Uehara** ***et al.*** **(2008).** The ROC curves obtained from different cross-validation folds were averaged based on the thresholds for class discrimination and drawn separately for each of the six classification methods (SVM, KNN, PAM, Random Forest, Weighted Voting and Naive Bayes). These classifiers were trained on **(A)** the original signature reported by the authors, **(B)** the signature inferred using our EFS method or **(C)** the signature obtained from our SR method.
(PDF)

Figure S4 **Evaluation of EFS-based and SR-based signatures on data from Nie** ***et al.*** The ROC curves obtained from different cross-validation folds were averaged based on the thresholds for class discrimination and drawn separately for each of the six classification methods (SVM, KNN, PAM, Random Forest, Weighted Voting and Naive Bayes). These classifiers were trained on **(A)** the original signature reported by the authors, **(B)** the signature inferred using our EFS method or **(C)** the signature obtained from our SR method.
(PDF)

Figure S5 **Evaluation of EFS-based and SR-based signatures on data from Fielden** ***et al.*** The ROC curves obtained from different cross-validation folds were averaged based on the thresholds for class discrimination and drawn separately for each of the six classification methods (SVM, KNN, PAM, Random Forest, Weighted Voting and Naive Bayes). These classifiers were trained on **(A)** the original signature reported by the authors, **(B)** the signature inferred using our EFS method or **(C)** the signature obtained from our SR method.
(PDF)

Figure S6 **Evaluation of EFS-based and SR-based signatures on data from Auerbach** ***et al.*** The ROC curves obtained from different cross-validation folds were averaged based on the thresholds for class discrimination and drawn separately for each of the six classification methods (SVM, KNN, PAM, Random Forest, Weighted Voting and Naive Bayes). These classifiers were trained on **(A)** the original signature reported by the authors, **(B)** the signature inferred using our EFS method or **(C)** the signature obtained from our SR method.
(PDF)

Figure S7 **Toxicogenomics-based assessment of compound carcinogenicity using published signatures.** The heatmaps show the confidence scores obtained from classifiers which were trained on published signatures for NGC prediction and applied to assess the carcinogenic potential of genotoxic and undefined compounds. One heatmap is depicted for each signature. Rows represent classifiers and columns correspond to compounds. The color intensity indicates the confidence that a certain compound is carcinogenic (blue) or non-carcinogenic (green).
(PDF)

Figure S8 **PCA-based separation of compounds based on published signatures.** Shown are the PCA-transformed expression profiles observed for different compounds in rat liver samples after treatment for 14 days. For this purpose, the compounds were represented by a vector composed of the fold-changes of the informative genes used in a certain published mRNA signature. PCA was then used to reduce the dimensionality of these vectors to the two principal components. Each of the plots corresponds to a certain signature (see titles). The dots correspond to different compounds, which are colored according to the compound class (see legend). Clusters of NGCs and NCs, respectively, are indicated by polygons drawn around the respective compounds. The compounds WY, MP and MCT were considered as undefined, due to ambiguous outcomes of published studies.
(PDF)

Figure S9 **Expression profiles of SR signature genes.** The heatmap depicts the expression profiles of the top 10 informative genes from the SR signature. The rows correspond to genes and the columns to treatment groups. Red indicates upregulation and green indicates downregulation. The annotated classes of the compounds are represented by the color bar on top. The boundaries between compound classes are highlighted by black lines.
(PDF)

Table S1 **Excel file with published and predicted signatures.** This file contains the Affymetrix probesets, gene symbols, Entrez Gene IDs and descriptions of all genes contained in published or novel signatures in tabular format. If necessary, custom probesets were mapped to the corresponding Affymetrix IDs via the associated gene symbols.
(XLS)

Table S2 **Excel file with pathways enriched in signatures.** This file contains the KEGG pathways enriched among the genes of each published or novel signature in tabular format. For each pathway the corresponding KEGG identifier, name and the number of deregulated genes is given. The significance of the enrichment was measured by means of FDR-corrected *p*-values (*q*-values) using a cutoff of *q* <0.05.
(XLS)
