# Supplementary figures and images for: Evaluation of Toxicogenomics Approaches for Assessing the Risk of Nongenotoxic Carcinogenicity in Rat Liver
Source: PLoS One. 2014 May 14;9(5):e97678. doi: 10.1371/journal.pone.0097678 (PMC4020844; doi:10.1371/journal.pone.0097678)

# Comparison on dataset by Ellinger *et al.*

A

Original signature

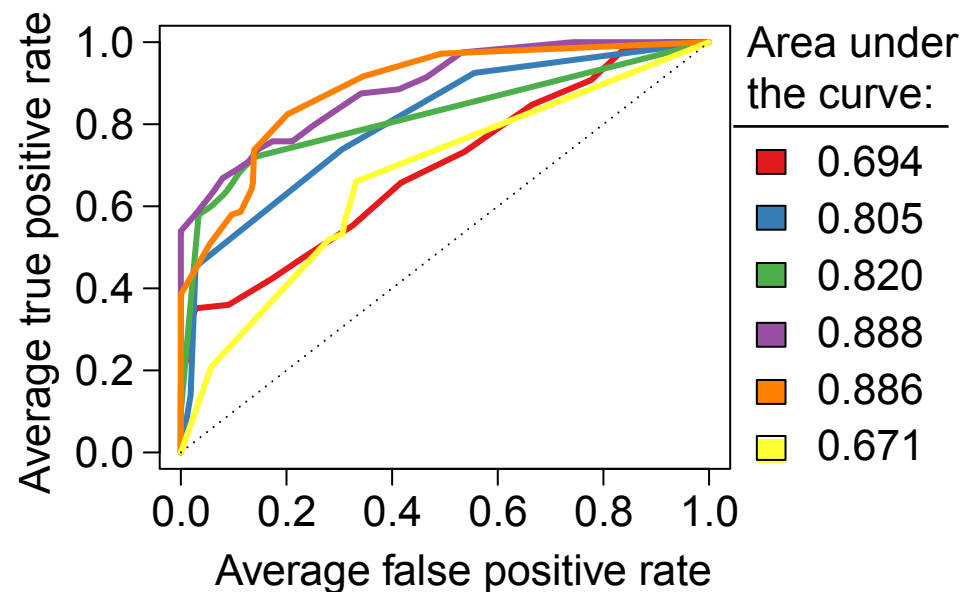

B

EFS signature

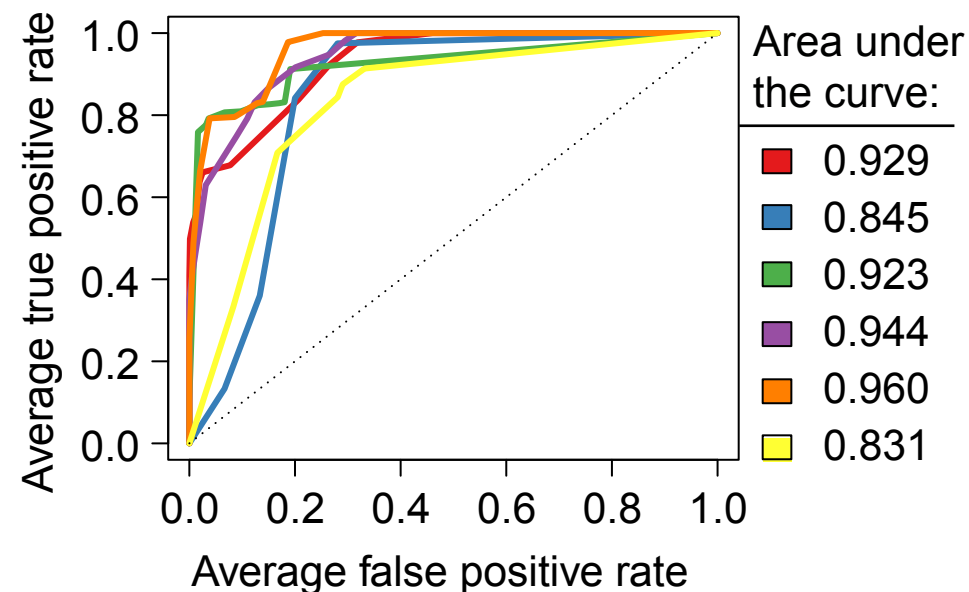

C

SR signature

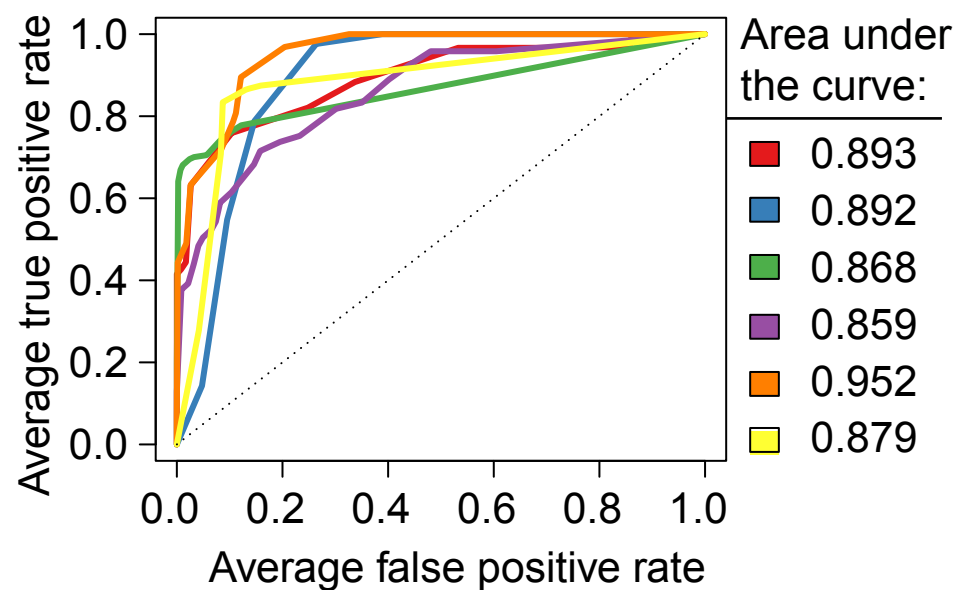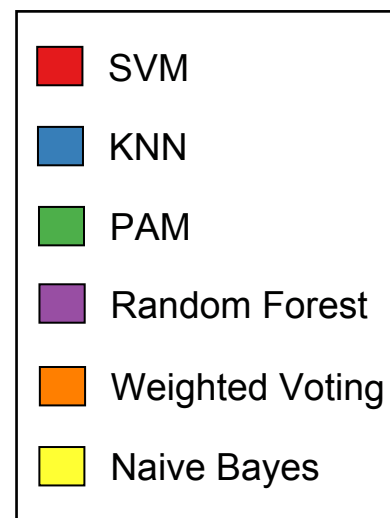

Supplement: Figure S1 — Evaluation of EFS-based and SR-based signatures on dataset from Ellinger et al. The ROC curves obtained from different cross-validation folds were averaged based on the thresholds for class discrimination and drawn separately for each of the six classification methods (SVM, KNN, PAM, Random Forest, Weighted Voting and Naive Bayes). These classifiers were trained on (A) the original signature reported by the authors, (B) the signature inferred using our EFS method or (C) the signature obtained from our SR method. (PDF) [file pone.0097678.s001.pdf]

# Comparison on dataset by Uehara *et al.* (2011)

A

Original signature

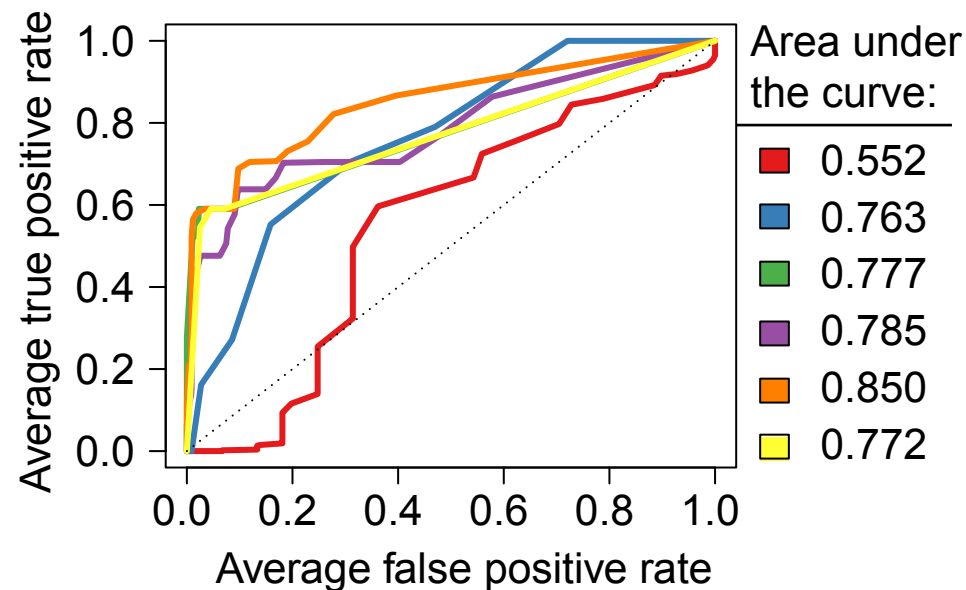

B

EFS signature

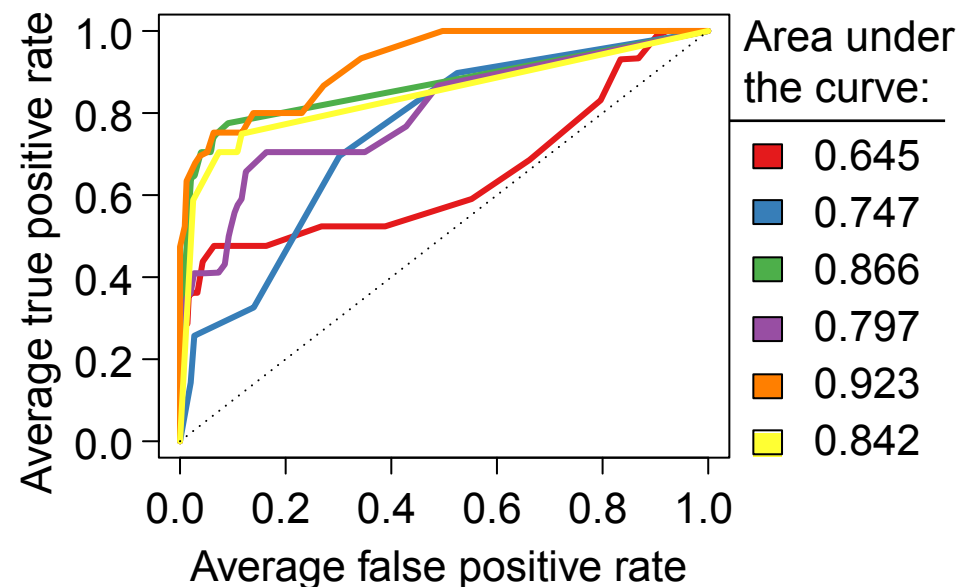

C

SR signature

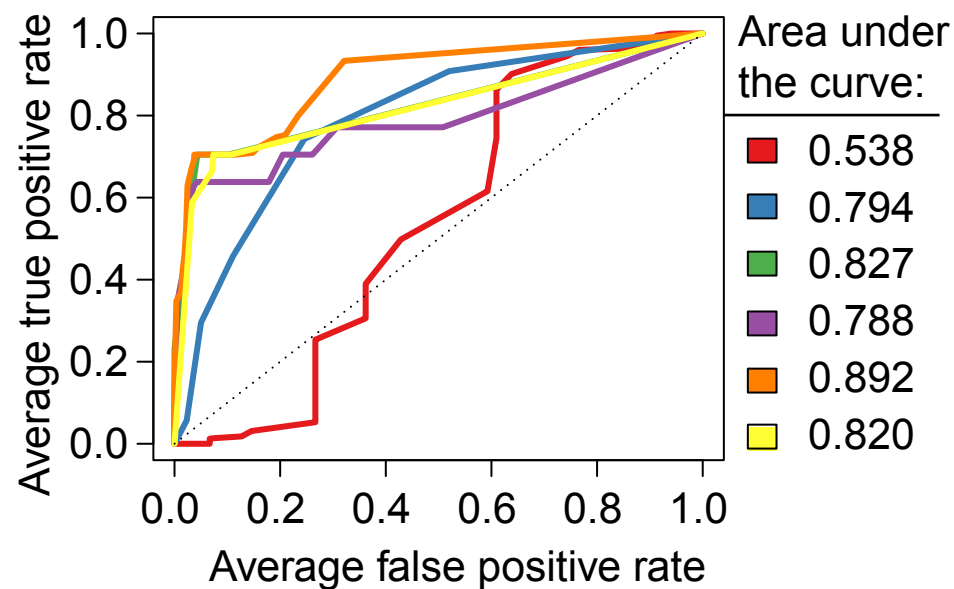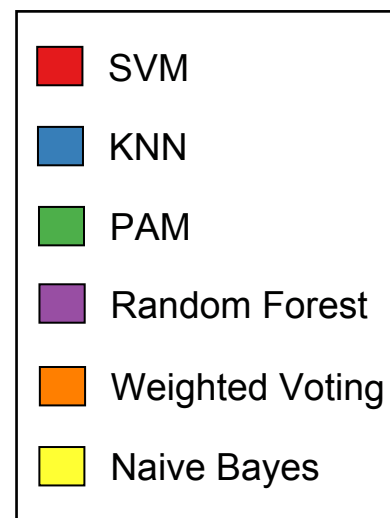

Supplement: Figure S2 — Evaluation of EFS-based and SR-based signatures on data from Uehara et al. (2011). The ROC curves obtained from different cross-validation folds were averaged based on the thresholds for class discrimination and drawn separately for each of the six classification methods (SVM, KNN, PAM, Random Forest, Weighted Voting and Naive Bayes). These classifiers were trained on (A) the original signature reported by the authors, (B) the signature inferred using our EFS method or (C) the signature obtained from our SR method. (PDF) [file pone.0097678.s002.pdf]

# Comparison on dataset by Uehara *et al.* (2008)

A

Original signature

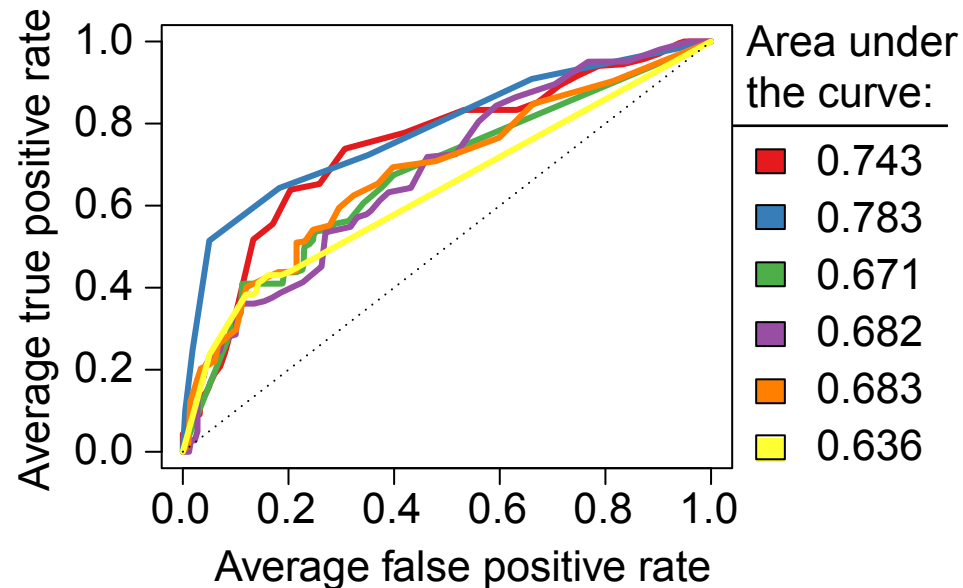

B

EFS signature

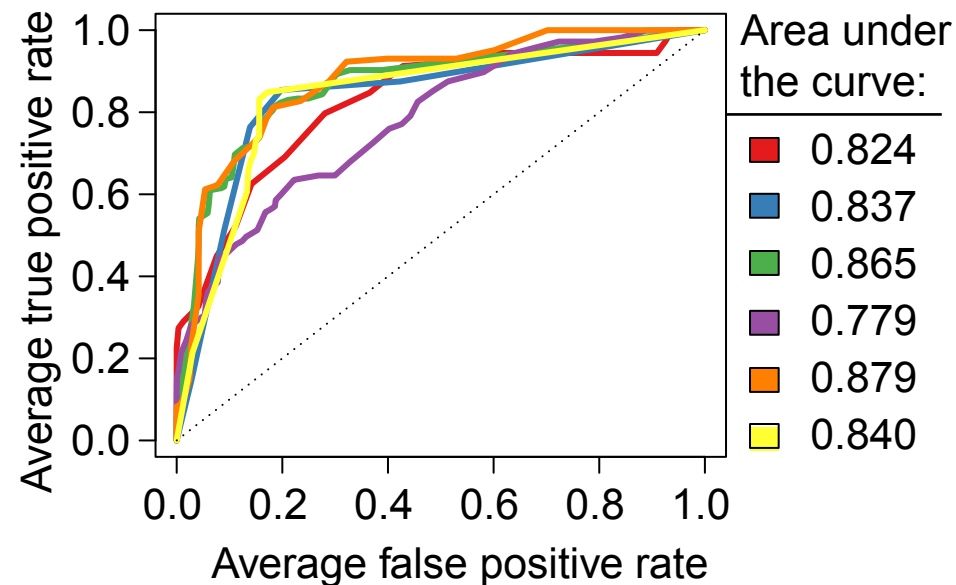

C

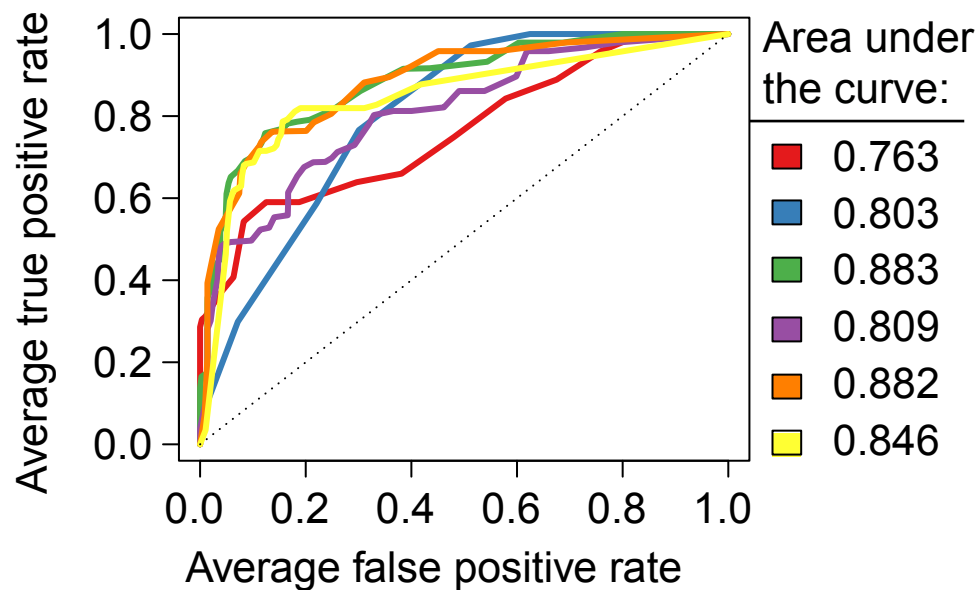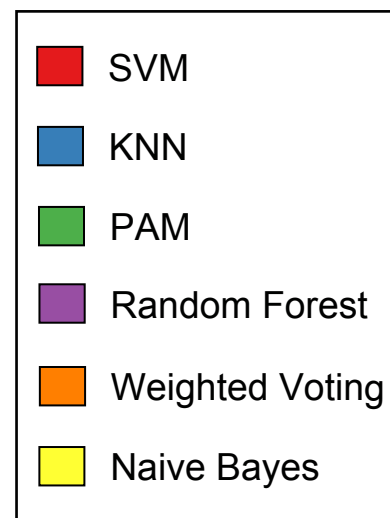

Supplement: Figure S3 — Evaluation of EFS-based and SR-based signatures on data from Uehara et al. (2008). The ROC curves obtained from different cross-validation folds were averaged based on the thresholds for class discrimination and drawn separately for each of the six classification methods (SVM, KNN, PAM, Random Forest, Weighted Voting and Naive Bayes). These classifiers were trained on (A) the original signature reported by the authors, (B) the signature inferred using our EFS method or (C) the signature obtained from our SR method. (PDF) [file pone.0097678.s003.pdf]

# Comparison on dataset by Nie et al.

A

Original signature

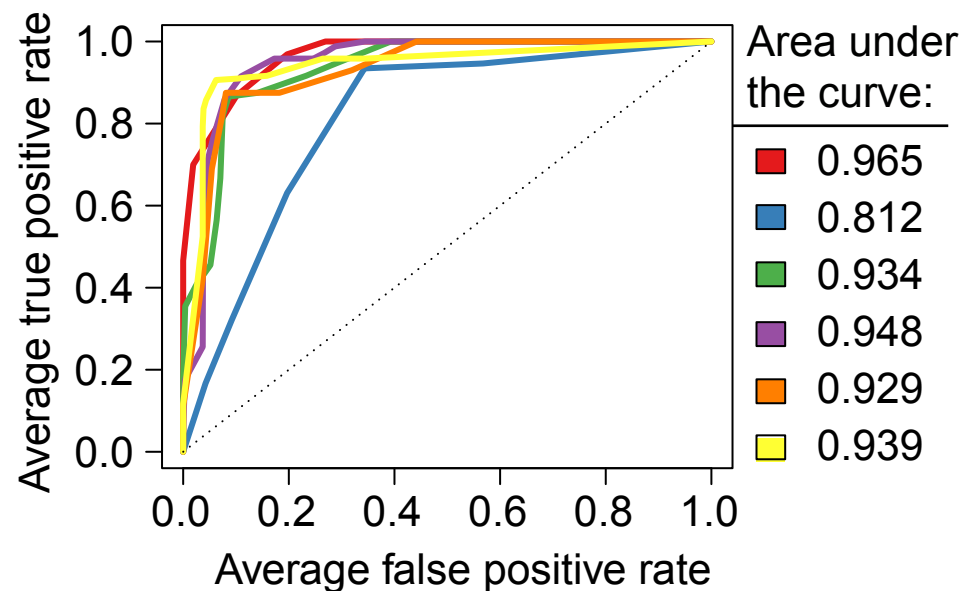

B

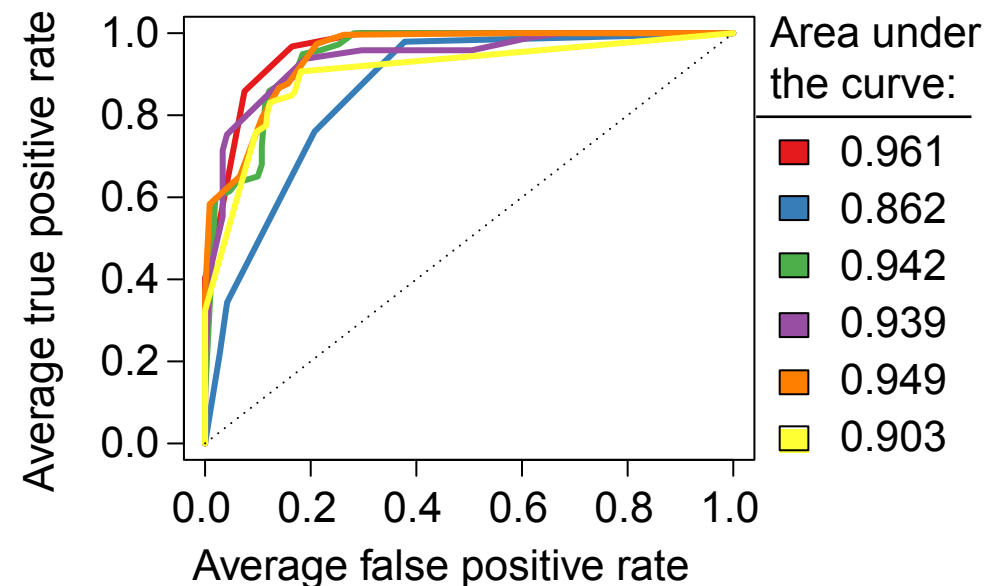

C

SR signature

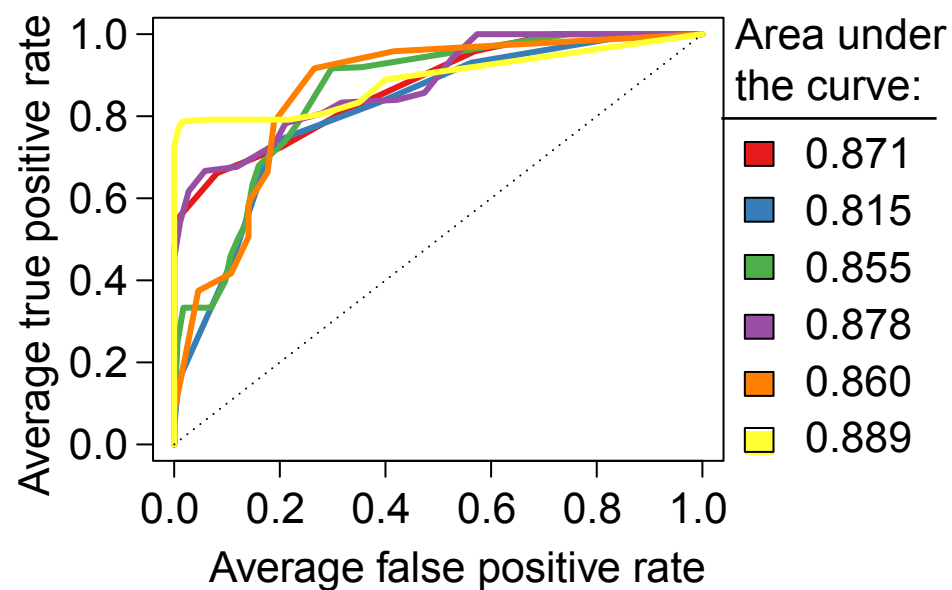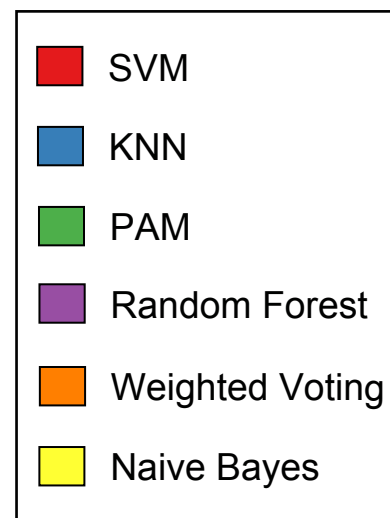

Supplement: Figure S4 — Evaluation of EFS-based and SR-based signatures on data from Nie et al. The ROC curves obtained from different cross-validation folds were averaged based on the thresholds for class discrimination and drawn separately for each of the six classification methods (SVM, KNN, PAM, Random Forest, Weighted Voting and Naive Bayes). These classifiers were trained on (A) the original signature reported by the authors, (B) the signature inferred using our EFS method or (C) the signature obtained from our SR method. (PDF) [file pone.0097678.s004.pdf]

# Comparison on dataset by Fielden *et al.*

A

Original signature

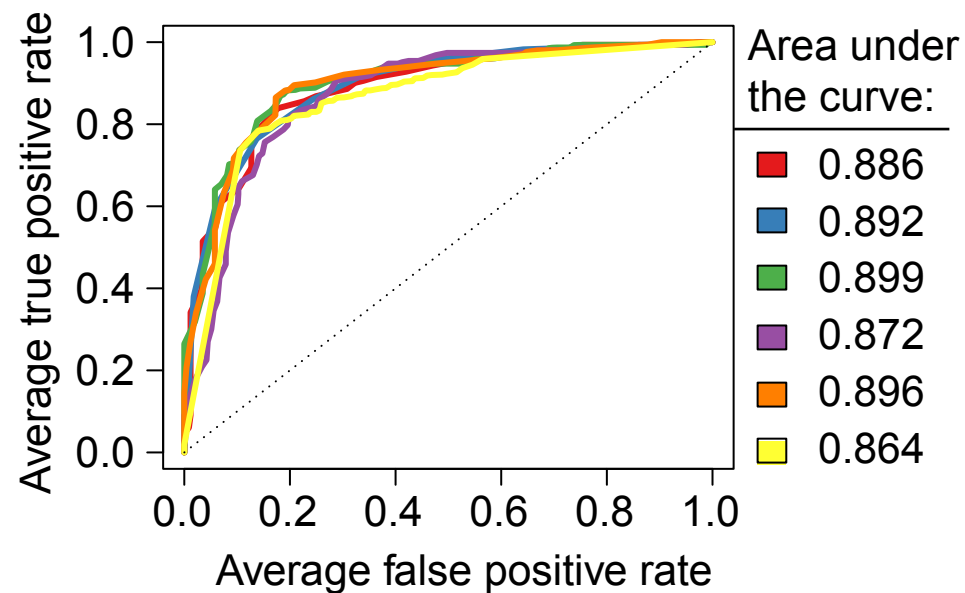

B

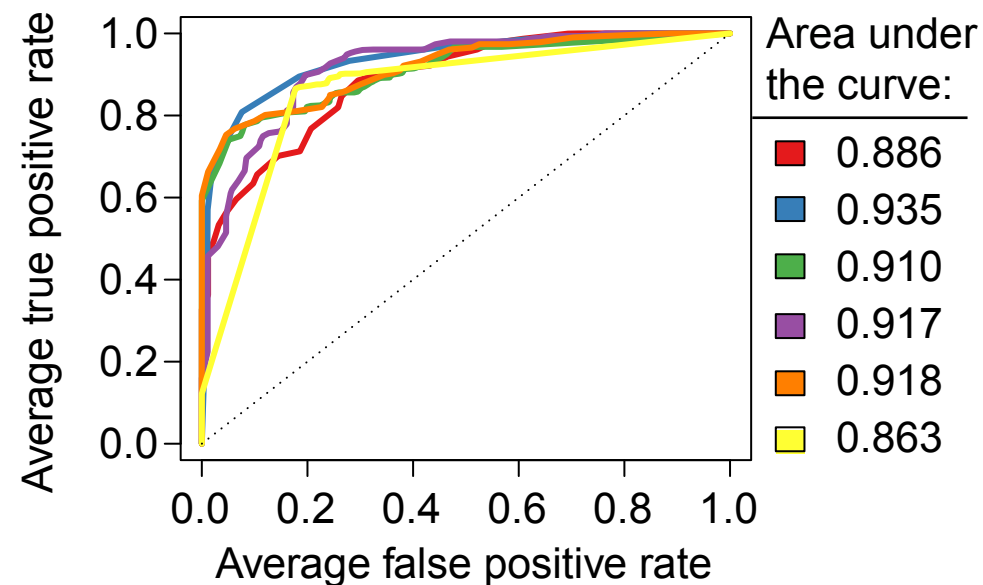

C

SR signature

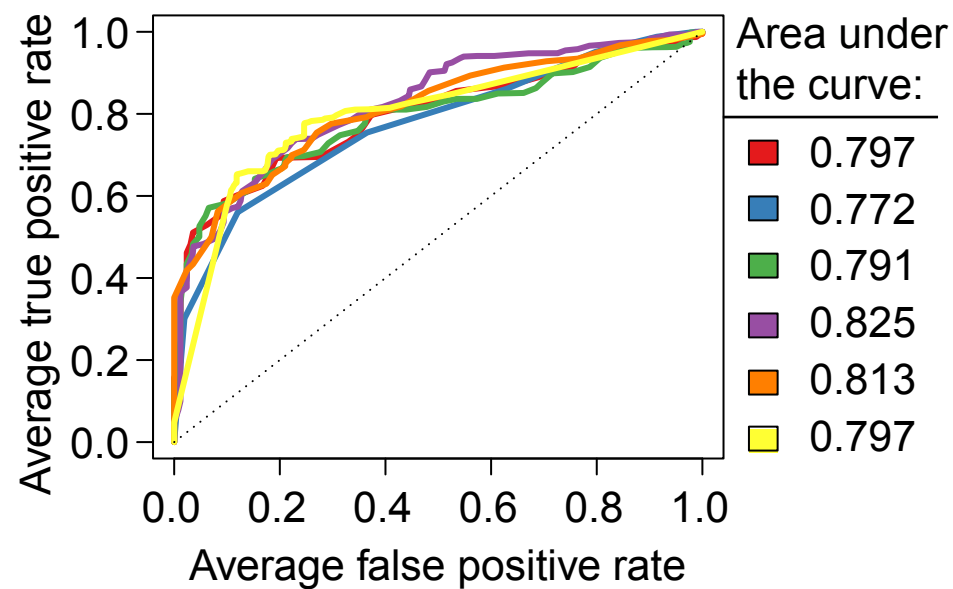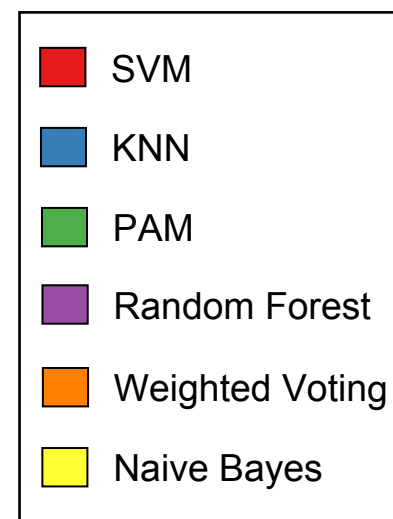

Supplement: Figure S5 — Evaluation of EFS-based and SR-based signatures on data from Fielden et al. The ROC curves obtained from different cross-validation folds were averaged based on the thresholds for class discrimination and drawn separately for each of the six classification methods (SVM, KNN, PAM, Random Forest, Weighted Voting and Naive Bayes). These classifiers were trained on (A) the original signature reported by the authors, (B) the signature inferred using our EFS method or (C) the signature obtained from our SR method. (PDF) [file pone.0097678.s005.pdf]

# Comparison on dataset by Auerbach et al.

A

Original signature

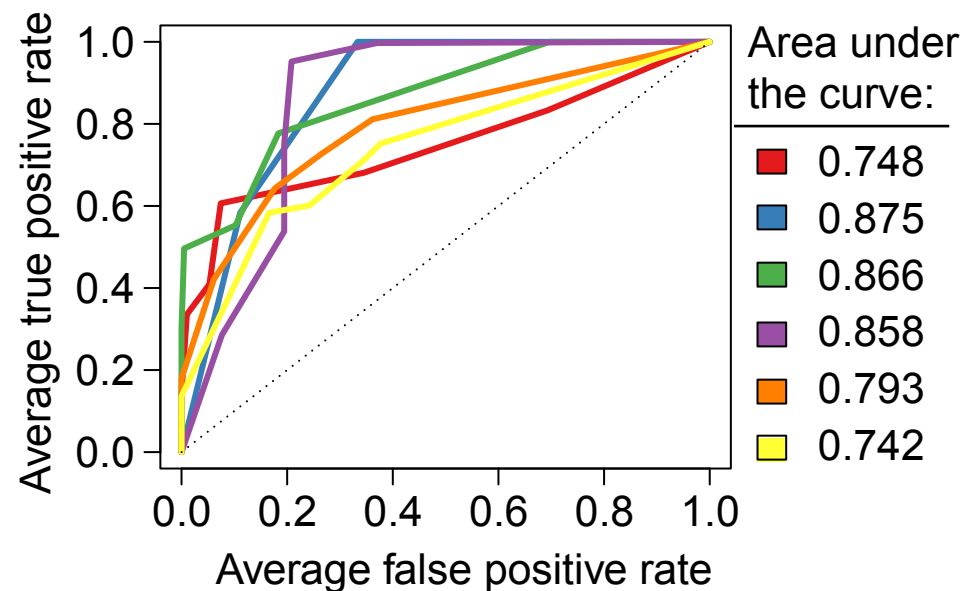

B

EFS signature

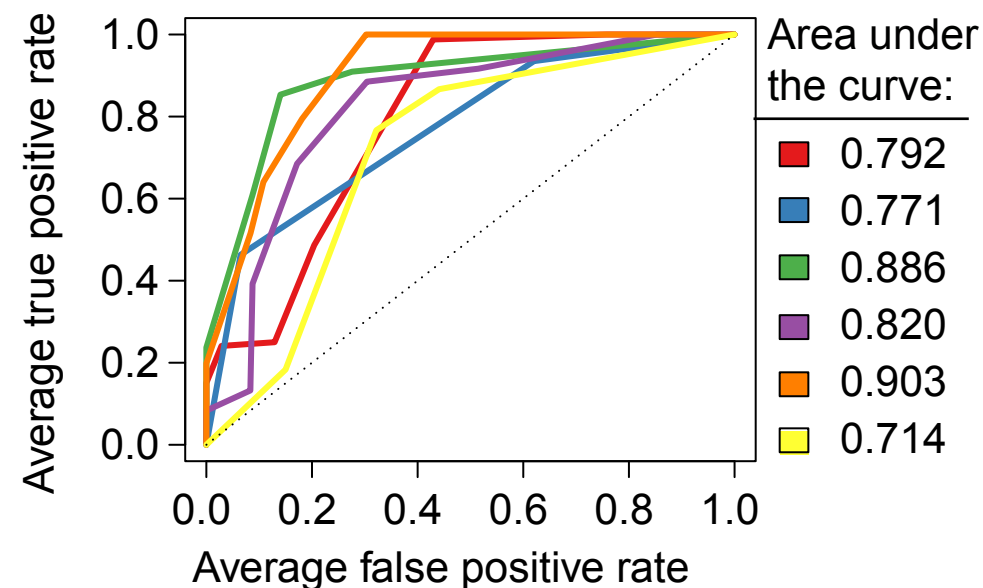

C

SR signature

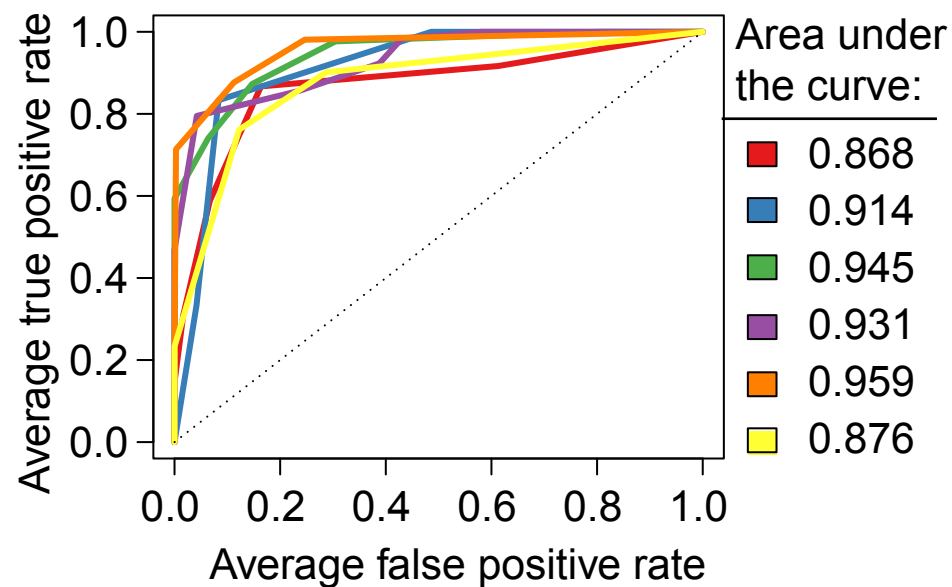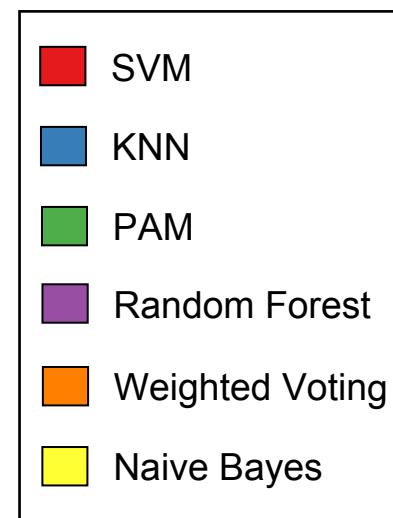

Supplement: Figure S6 — Evaluation of EFS-based and SR-based signatures on data from Auerbach et al. The ROC curves obtained from different cross-validation folds were averaged based on the thresholds for class discrimination and drawn separately for each of the six classification methods (SVM, KNN, PAM, Random Forest, Weighted Voting and Naive Bayes). These classifiers were trained on (A) the original signature reported by the authors, (B) the signature inferred using our EFS method or (C) the signature obtained from our SR method. (PDF) [file pone.0097678.s006.pdf]

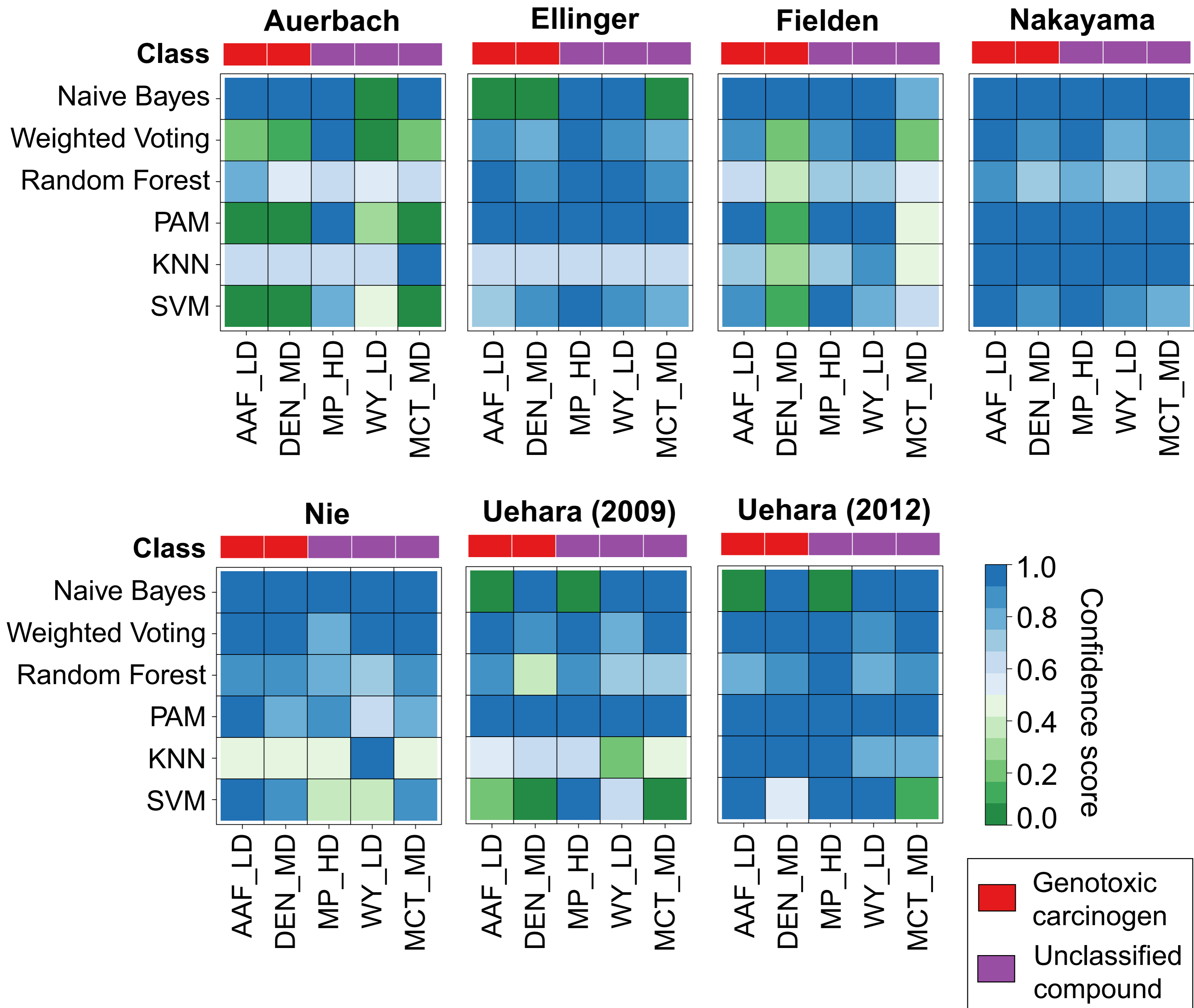

Supplement: Figure S7 — Toxicogenomics-based assessment of compound carcinogenicity using published signatures. The heatmaps show the confidence scores obtained from classifiers which were trained on published signatures for NGC prediction and applied to assess the carcinogenic potential of genotoxic and undefined compounds. One heatmap is depicted for each signature. Rows represent classifiers and columns correspond to compounds. The color intensity indicates the confidence that a certain compound is carcinogenic (blue) or non-carcinogenic (green). (PDF) [file pone.0097678.s007.pdf]

# Heatmap plot of SR signature

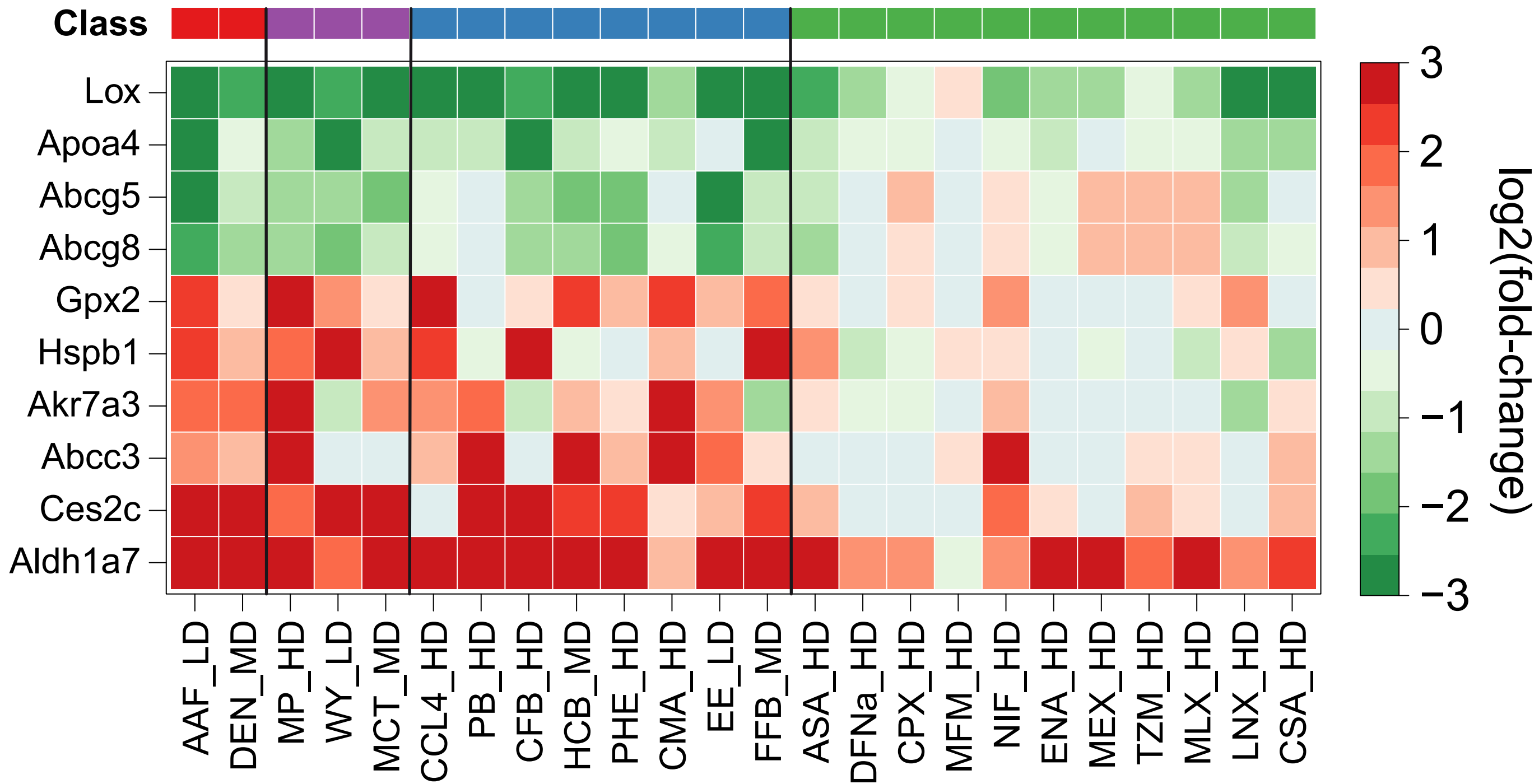

Supplement: Figure S9 — Expression profiles of SR signature genes. The heatmap depicts the expression profiles of the top 10 informative genes from the SR signature. The rows correspond to genes and the columns to treatment groups. Red indicates upregulation and green indicates downregulation. The annotated classes of the compounds are represented by the color bar on top. The boundaries between compound classes are highlighted by black lines. (PDF) [file pone.0097678.s009.pdf]
