# Supplementary material for: Evaluation of Toxicogenomics Approaches for Assessing the Risk of Nongenotoxic Carcinogenicity in Rat Liver
Source: PLoS One. 2014 May 14;9(5):e97678. doi: 10.1371/journal.pone.0097678 (PMC4020844; doi:10.1371/journal.pone.0097678)
Supplement: Figure S8 — PCA-based separation of compounds based on published signatures. Shown are the PCA-transformed expression profiles observed for different compounds in rat liver samples after treatment for 14 days. For this purpose, the compounds were represented by a vector composed of the fold-changes of the informative genes used in a certain published mRNA signature. PCA was then used to reduce the dimensionality of these vectors to the two principal components. Each of the plots corresponds to a certain signature (see titles). The dots correspond to different compounds, which are colored according to the compound class (see legend). Clusters of NGCs and NCs, respectively, are indicated by polygons drawn around the respective compounds. The compounds WY, MP and MCT were considered as undefined, due to ambiguous outcomes of published studies. (PDF) [file pone.0097678.s008.pdf]

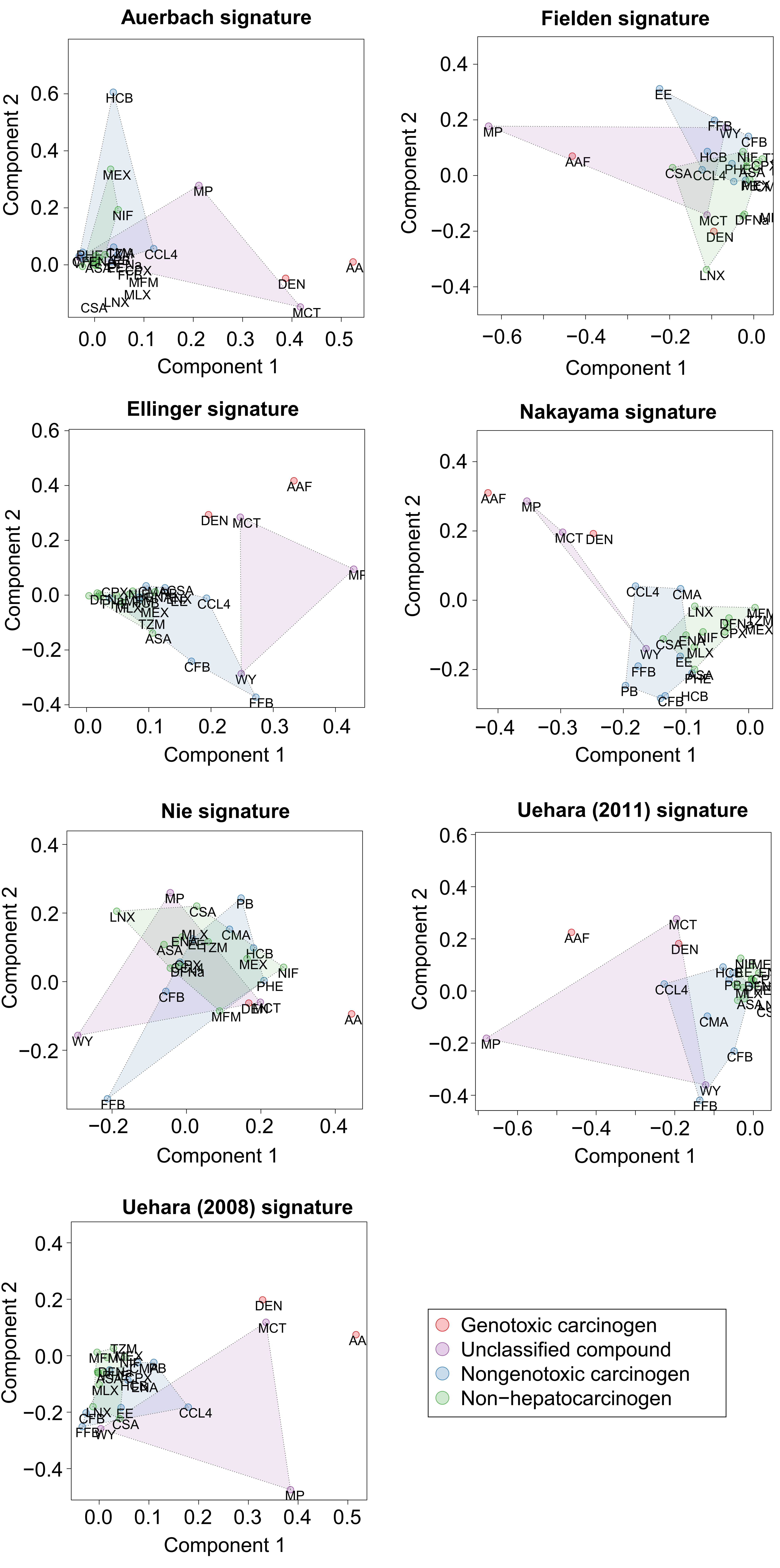

Auerbach signature
Component 2
Component 1
HCB
MEX
NIF
MP
CCL4
DEN
AA
MFM
MLX
LNX
CSA
MCT
Fielden signature
Component 2
Component 1
EE
FFB
WY
CFB
MP
AAF
HCB
NIF
CSA
CCL4
ASA
MCT
DFNa
DEN
LNX
Ellinger signature
Component 2
Component 1
AAF
DEN
MCT
CSA
CCL4
MEX
TZM
ASA
CFB
WY
FFB
Nakayama signature
Component 2
Component 1
AAF
MP
MCT
DEN
CCL4
CMA
LNX
CSA
NIF
MLX
WY
EE
FFB
PHE
PB
HCB
CFB
Nie signature
Component 2
Component 1
MP
CSA
PB
LNX
MLX
CMA
ASA
TZM
HCB
MEX
NIF
CPX
DFNa
PHE
CFB
MCT
MFM
AA
WY
FFB
Uehara (2011) signature
Component 2
Component 1
AAF
MCT
DEN
CCL4
CMA
ASA
MP
CFB
WY
FFB
Uehara (2008) signature
Component 2
Component 1
DEN
MCT
AA
TZM
LNX
EE
CFB
CSA
CCL4
FFB
WY
MP
Genotoxic carcinogen
Unclassified compound
Nongenotoxic carcinogen
Non−hepatocarcinogen
